# Capicua regulates the development of adult-born neurons in the hippocampus

**DOI:** 10.1038/s41598-021-91168-5

**Published:** 2021-06-03

**Authors:** Brenna Hourigan, Spencer D. Balay, Graydon Yee, Saloni Sharma, Qiumin Tan

**Affiliations:** 1 Department of Cell Biology, University of Alberta, Edmonton, T6J 2H7 Canada; 2 Present Address: Research Institute of Molecular Pathology, Vienna Biocenter, Campus-Vienna-Biocenter 1, 1030 Vienna, Austria

**Keywords:** Cell biology, Developmental biology, Molecular biology, Neuroscience

## Abstract

New neurons continuously arise from neural progenitor cells in the dentate gyrus of the adult hippocampus to support ongoing learning and memory formation. To generate functional adult-born neurons, neural progenitor cells proliferate to expand the precursor cell pool and differentiate into neurons. Newly generated cells then undergo postmitotic maturation to migrate to their final destination and develop elaborate dendritic branching, which allows them to receive input signals. Little is known about factors that regulate neuronal differentiation, migration, and dendrite maturation during adult hippocampal neurogenesis. Here, we show that the transcriptional repressor protein capicua (CIC) exhibits dynamic expression in the adult dentate gyrus. Conditional deletion of *Cic* from the mouse dentate gyrus compromises the adult neural progenitor cell pool without altering their proliferative potential. We further demonstrate that the loss of *Cic* impedes neuronal lineage development and disrupts dendritic arborization and migration of adult-born neurons. Our study uncovers a previously unrecognized role of CIC in neurogenesis of the adult dentate gyrus.

## Introduction

**Figure 1:**
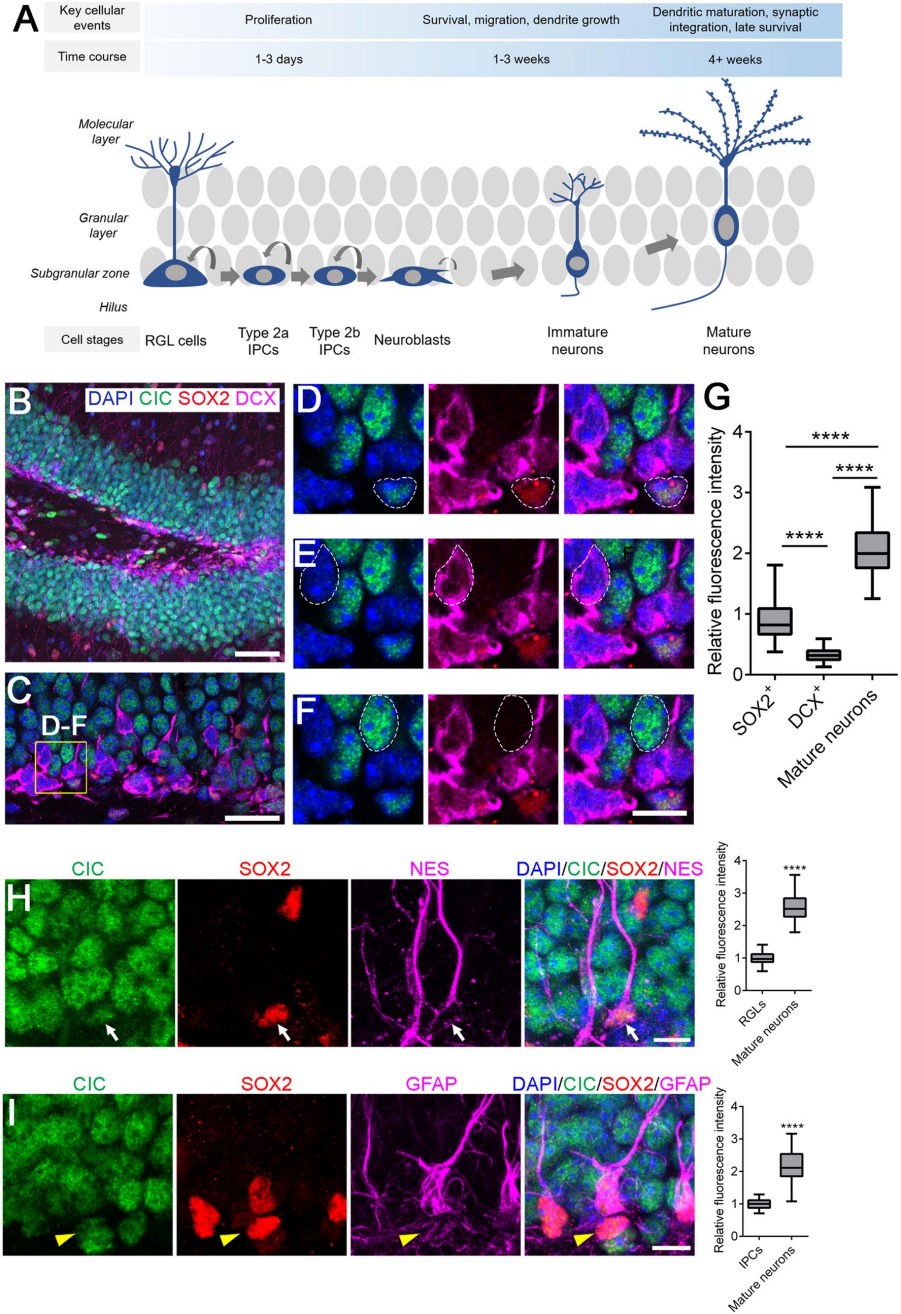
CIC expression along the neurogenic lineage in the adult dentate gyrus. (**A**) A schematic of adult hippocampal neurogenesis. The process of adult hippocampal neurogenesis encompasses four phases with six developmental milestones identifiable through cell morphology and expression of specific protein markers. Radial glia-like (RGL) cells are mostly quiescent stem cells that reside in the subgranular zone. Upon activation, RGL cells give rise to rapidly amplifying type 2a and then type 2b intermediate progenitor cells (IPCs). IPCs differentiate to neuroblasts which eventually exit the cell cycle to become immature and then mature granule neurons. SOX2 and GFAP are expressed by RGL and type 2a cells. DCX starts to be expressed by type 2b cells but is turned off when neurons become mature. (**B**) A representative image showing CIC, SOX2 and DCX expression in the dentate gyrus of 8-week-old wildtype mice. Scale bar = 50 µm. (**C**) CIC, SOX2 and DCX expression in the neurogenic subgranular zone. Scale bar = 25 µm. (**D**) During adult hippocampal neurogenesis, SOX2^+^ neural progenitor cells express moderate levels of CIC. (**E**) Minimal CIC expression is observed in DCX^+^ cells. (**F**) Mature neurons have the highest levels of CIC. Scale bar = 10 µm. (**G**) Quantification of relative fluorescence intensity of CIC immunostaining in different cell stages. N = 60 cells from a total of three 8-week-old wildtype mice. (**H**) CIC is expressed in SOX2^+^ NES^+^ RGL cells. Arrow points to a RGL cell. Scale bar = 10 µm. Quantification is shown to the right. N = 59 RGL cells and 80 mature neurons from a total of four 12-week-old control mice. (**I**) CIC is expressed in SOX2^+^ GFAP^-^ IPCs. Arrowhead points to an IPC. Scale bar = 10 µm. Quantification is shown to the right. N = 15 IPCs and 51 mature neurons from a total of three 12-week-old control mice. Data are presented in box-and-whisker plots, where centre lines represent medians, box limits represent interquartile ranges, and whiskers represent minimum to maximum data ranges. Statistical analyses were performed with one-way ANOVA with Tukey’s post hoc test (in **G**) or two-tailed Student’s *t*-test (**H**, **I**). ****P < 0.0001.

New neurons are continuously generated in the adult mammalian hippocampus. These adult-born neurons play important roles in learning, memory formation and mood regulation^1^. Quiescent radial glial-like (RGL) neural progenitor cells, which reside in the subgranular zone (SGZ) of the hippocampal dentate gyrus, develop into mature granule neurons through four phases: a progenitor cell phase, an early survival phase, a postmitotic maturation phase, and a late survival/maturation phase^2^. This entire process takes about 6–8 weeks and encompasses six developmental milestones which can be identified based on molecular markers and cell morphology (Fig. 1A). The progenitor cell phase is the shortest, lasting only 1–3 days, and serves to expand the pool of rapidly amplifying intermediate progenitor cells. Most of these newly generated cells, however, are eliminated within days after they exit the cell cycle during the early survival phase. The few surviving cells will proceed to the postmitotic maturation phase, when they migrate tangentially and radially into the granule cell layer and assume a polarized morphology by extending an elaborate dendritic tree toward the molecular layer and projecting an axon into the hilus^3^. Adult-born neurons undergo further dendritic maturation and fine-tuning of their electrophysiological properties during the late survival/maturation phase^2–4^.

Extensive studies of adult hippocampal neurogenesis have focused on the progenitor cell phase; these studies have revealed that progenitor cell maintenance is controlled by myriads of extrinsic and intrinsic signals and the activities of a coordinated network of transcription factors^5^. In contrast, little is known about the mechanisms underlying neuronal differentiation, survival, and maturation, including the selective vulnerability to cell death, the initiation and termination of migration, and the establishment of dendritic architecture. Clues to answer these questions may come from embryonic and early postnatal neurogenesis. Recent lineage tracing and single-cell transcriptomic studies demonstrate that adult and developmental dentate gyrus neurogenesis are one continuous process that share a common cellular origin and conserved molecular properties^6,7^. Moreover, many transcription factors important for embryonic cortical and hippocampal development also play a role in adult dentate gyrus neurogenesis^5^. Therefore, we asked whether transcriptional regulators of neuronal migration and maturation in the developing brain may be required for similar processes in the adult brain.

To further investigate this possibility, we turned to capicua (CIC), a transcriptional repressor first identified in *Drosophila* embryos as specifying terminal regions—head and tail, hence the Catalan name meaning “head–and–tail”^8^. Beyond embryonic patterning, CIC plays important roles in many other aspects of *Drosophila* development, including wing vein specification, intestinal stem cell proliferation, and organ growth^9,10^. The function of CIC in mammalian development is less well-understood, but CIC has emerged as an important regulator of neuronal maturation during early brain development. Pan-neural (*Nestin-Cre*) *Cic* knockout mice die by weaning age with prominent maturation defects in cortical neurons and dentate gyrus granule neurons^11^. Neocortical excitatory neurons in the *Emx1-Cre Cic* knockout mice, which lack *Cic* in forebrain excitatory neurons and glia, have reduced post-mitotic survival and the surviving neurons develop abnormal dendritic arborization^12^. These findings highlight the importance of CIC in neuronal maturation and dendrite development during early postnatal brain development. However, whether CIC is similarly required for neurons to mature during adult neurogenesis remains unknown.

To understand the role of CIC in the adult brain, we evaluated CIC expression and its role in adult hippocampal neurogenesis. We find that CIC is required for maintenance of the adult progenitor cell pool, proper neuronal lineage development, as well as development of dendritic branching of new neurons. Loss of CIC also disrupted normal migration of some adult-born neurons. Our study uncovers a previously unrecognized role of CIC in adult hippocampal neurogenesis and increases our knowledge of transcription factors that regulate adult-born neuron development in the dentate gyrus.

## Results

### CIC shows a dynamic expression pattern during the progression from neural progenitor cells to mature granule neurons

We first sought to map CIC expression in the adult mouse brain. Immunostaining revealed that CIC is widely expressed, with highest expression in the dentate gyrus of the hippocampus (Supplementary Fig. S1), where neurogenesis takes place. There are several cell types contained in the dentate gyrus (Fig. 1A). Quiescent radial glia-like (RGL) cells reside in the subgranular zone; upon activation, RGL cells differentiate into rapidly amplifying type 2a/b intermediate progenitor cells (IPCs)^2^. IPCs give rise to neuroblasts that eventually exit the cell cycle to become immature and then mature granule neurons that localize to the granule cell layer^13^. To determine which of these cell types express CIC, we co-immunostained for CIC and two specific markers, SOX2 and DCX, which labelled two broad, largely non-overlapping cell stages in the adult dentate gyrus (Fig. 1B–G). We observed nuclear CIC staining in the SGZ among neural progenitors (SOX2^+^ RGL cells and type 2a IPCs) (Fig. 1D). DCX^+^ neuroblasts/immature neurons showed a minimal level of CIC (Fig. 1E). Nonetheless, CIC expression in DCX^+^ cells was detectable under high laser power and detector gain with confocal microscopy (Supplementary Fig. S2). As DCX^+^ cells progressed to mature granule neurons, which were classified as cells in the granular layer negative for SOX2 and DCX, CIC was re-expressed and reached the highest levels in the nucleus (Fig. 1F, quantification in 1G). We further determined whether CIC was expressed in distinct SOX2^+^ progenitor populations. Our results showed that, compared to its levels in mature neurons, CIC was expressed at similar levels in SOX2^+^ NES^+^ RGL cells and SOX2^+^ GFAP^-^ IPCs (Fig. 1H, I).

In short, CIC is downregulated during the differentiation of SOX2^+^ neural progenitor cells into DCX^+^ cells, but its expression is restored and peaks as DCX^+^ cells mature into granule neurons. This dynamic expression pattern suggests a role of CIC in neuronal differentiation and maturation.

### The neural progenitor cell pool is reduced in the *Emx1-Cre Cic* knockout mice

To examine the function of CIC in adult hippocampal neurogenesis, we used the *Emx1-Cre; Cic*^*flox/flox*^ mice (herein referred to as the *Emx1-Cre Cic* knockout mice). The *Emx1-Cre* allele expresses the cre recombinase in forebrain neural progenitor cells starting from embryonic day 9.5, resulting in cre-mediated recombination in the developing and adult dentate gyrus^14^. In the present study, we investigated cellular defects of the dentate gyrus in the *Cic*^*flox/flox*^ (control), the *Emx1-Cre; Cic*^*flox/*+^ (conditional heterozygous), and the *Emx1-Cre; Cic*^*flox/flox*^ (conditional homozygous) mice. We first tested whether cre-mediated recombination in the adult dentate gyrus was complete in the *Emx1-Cre Cic* knockout mice. Immunostaining for CIC in the control and knockout mice showed that CIC was efficiently deleted from *Emx1*-lineage cells in all of the adult neurogenic cell stages, which included SOX2^+^ neural progenitor cells, DCX^+^ cells and mature granule neurons (Supplementary Fig. S3). CIC^+^ cells were occasionally found in the adult dentate gyrus of the knockout mice in what were likely non-*Emx1*-lineage cells.

**Figure 2:**
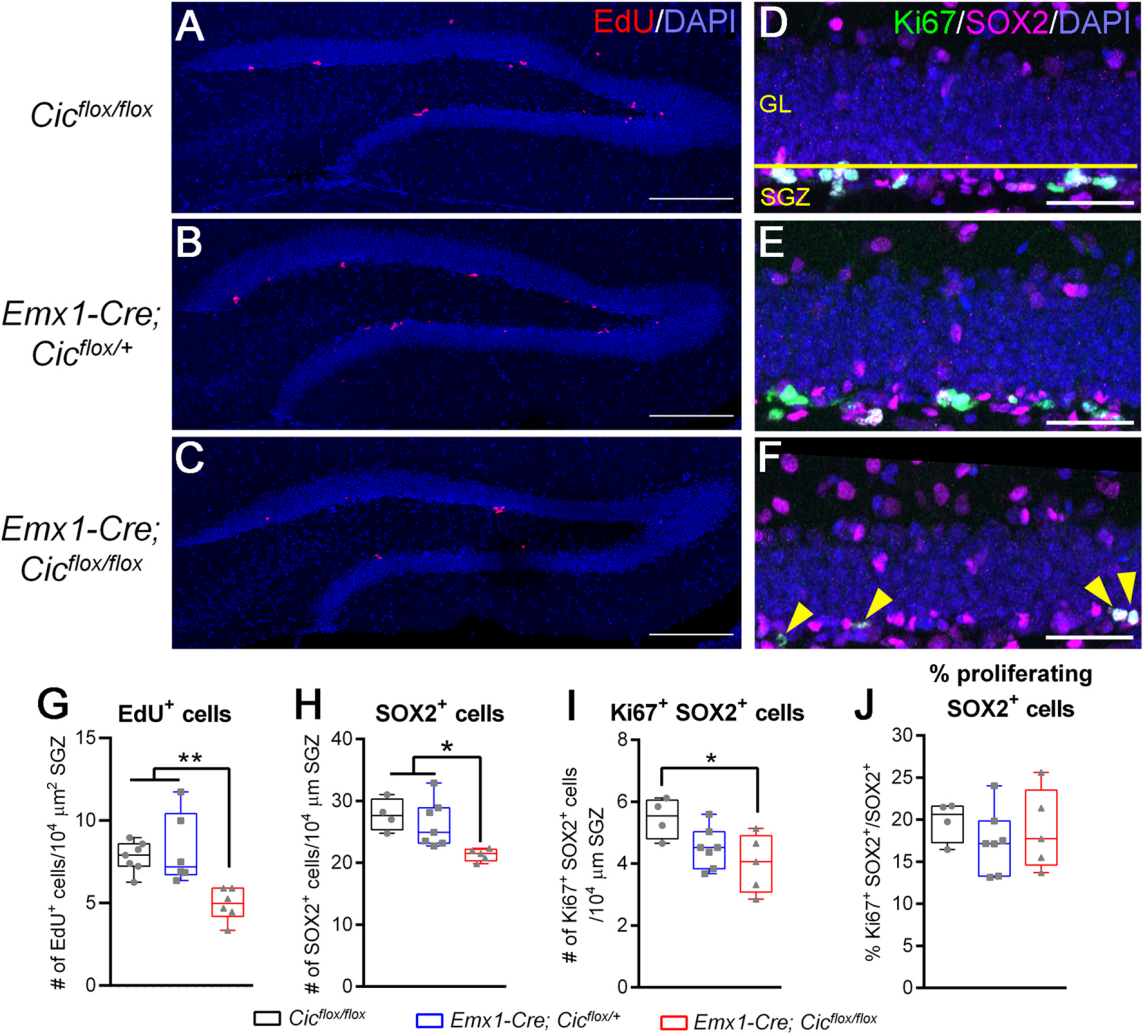
*Emx1-Cre Cic* knockout mice have a diminished pool of adult hippocampal neural progenitor cells. (**A**–**C**) Representative images of the dentate gyrus four hours post EdU injection showing staining for EdU (red) and DAPI (blue). EdU staining is mainly found in the neurogenic subgranular zone. Scale bars = 200 µm. (**D**–**F**) Representative images of the subgranular zone (SGZ) showing staining for Ki67 (green), SOX2 (magenta), and DAPI (blue). Yellow line in (D) indicates the boundary between the SGZ and the granule cell layer (GL). Yellow arrowheads in (F) point to cells double positive for SOX2 and Ki67. Scale bars = 50 µm. Quantifications of EdU^+^ cells (**G**), SOX2^+^ cells (**H**), Ki67^+^ SOX2^+^ cells (**I**), and the percentage of proliferating SOX2^+^ neural progenitor cells (**J**) are shown at the bottom. N = 4‒7 animals per group. Box-and-whisker plots show all data points; centre lines represent medians, box limits represent interquartile ranges, and whiskers represent minimum to maximum data ranges. Statistical analyses were performed with one-way ANOVA with Tukey’s post hoc test. *P < 0.05.

As the *Emx1-Cre* driver is turned on during early embryonic development, we first determined whether *Cic* deletion in these mice affected the formation of the adult neurogenic SGZ. We defined the SGZ as “a layer of cells expanding 5 μm into the hilus and 15 μm into the granular cell layer” as previously described^15^. Our analysis of 11-week-old control and *Emx1-Cre Cic* knockout mice showed that the size of the SGZ was not significantly altered in the knockout mice (Supplementary Fig. S4). We next assessed the effects of *Cic* deletion on neural progenitor cell proliferation. To this end, we used EdU (5-ethynyl-2′-deoxyuridine), a thymidine analogue, to label cells that had entered the S phase of the cell cycle during a 4-h period. The length of S phase for SGZ neural progenitor cells is 2–2.5 h and the total cell cycle length is about 22–24 h^16^. Therefore, this experimental paradigm labelled only those cells that had recently entered, and remained in, the cell cycle. We found a decrease in the number of EdU^+^ cells in the SGZ of the *Emx1-Cre Cic* knockout mice compared to the control mice (Fig. 2A–C, G). This reduction in the number of proliferating cells in the SGZ could be due to a decrease in the overall neural progenitor cell pool, a decline of the proliferation capacity of neural progenitor cells, or both. To tease apart these possibilities, we first compared the number of SGZ SOX2^+^ progenitor cells between the control and the *Emx1-Cre Cic* knockout mice. We observed fewer SOX2^+^ cells in the knockout mice, indicative of a smaller pool of progenitor cells (Fig. 2D–F, H). Our analyses further showed that both NES^+^ GFAP^+^ RGL cells and TBR2^+^ IPCs were significantly reduced in the knockout dentate gyrus (Supplementary Fig. S5). We next quantified the cells that were co-labelled with SOX2 and the proliferation marker Ki67, and found that fewer Ki67^+^ SOX2^+^ cells were present in the knockout mice (Fig. 2I). To determine the proliferation capacity of neural progenitor cells, we calculated the proportion of SOX2^+^ cells that were also co-labelled with Ki67. In the SGZ of control adult mice, about 20% of SOX2^+^ cells were actively dividing (Fig. 2J), which is comparable to findings from other studies^17–19^. The *Emx1-Cre Cic* knockout mice had a similar percentage of proliferating SOX2^+^ cells as the control mice, so the proliferation capacity of neural progenitor cells did not appear to be altered in the knockout mice. Altogether, these data suggest that, while the overall neural progenitor cell pool is diminished in the *Emx1-Cre Cic* knockout mice, their relative proliferation capacity is unaltered.

### CIC deletion does not affect the total number of DCX^+^ cells but impairs their development into mature neurons

**Figure 3:**
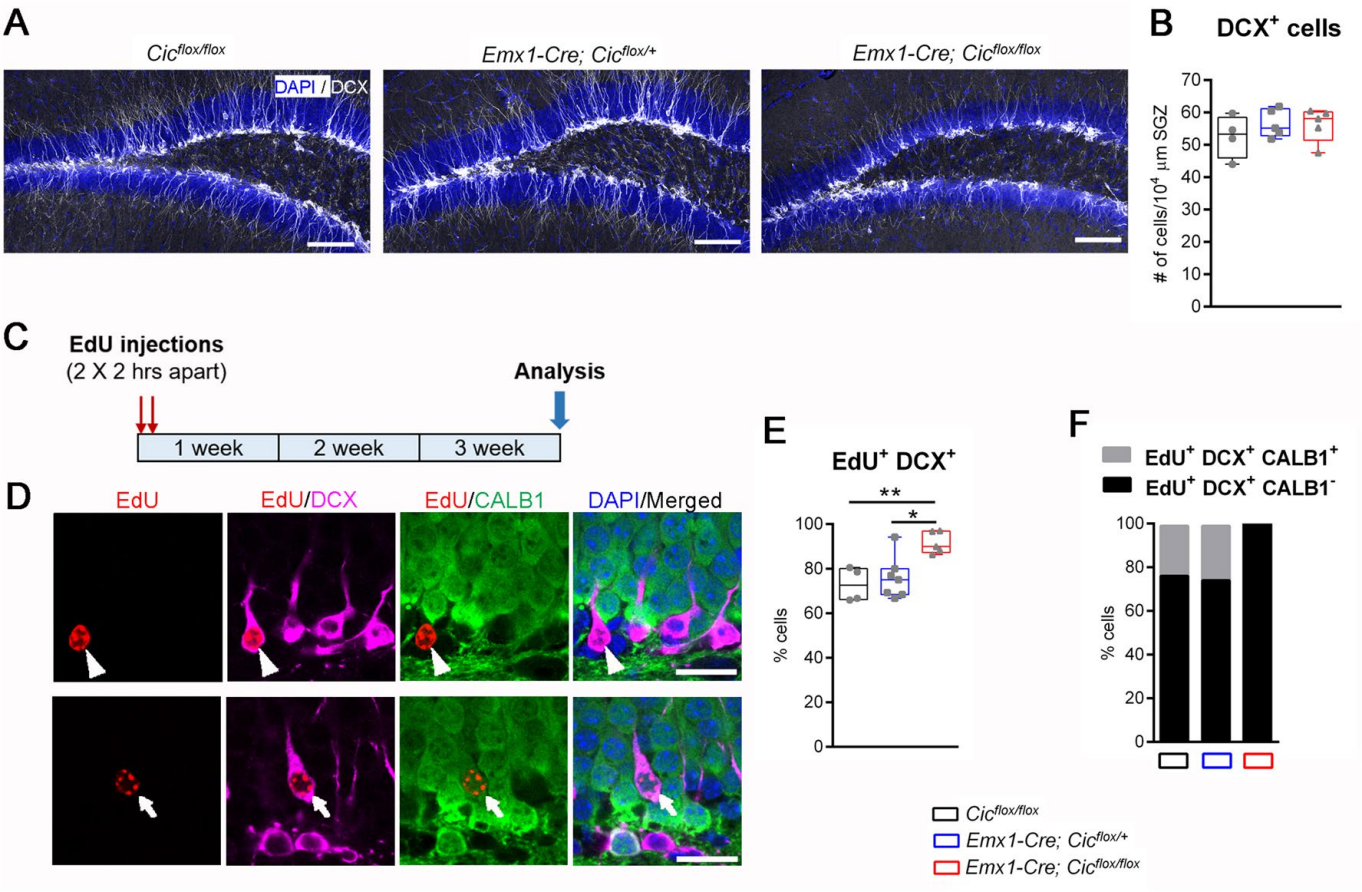
Impaired development of DCX^+^ cells in the *Emx1-Cre Cic* knockout mice. (**A**) Representative images of the dentate gyrus in the three mouse lines show severe dendritic morphology defects in DCX^+^ cells of the knockout mice. DAPI (blue) and DCX (grey). Scale bars = 100 µm. (**B**) Quantification of the total number of DCX^+^ cells in the three genotypes. No statistically significant difference was found. (**C**) A schematic of the EdU-labelling study. Mice were injected with two doses of EdU that were separated by two hours and were analysed three weeks post injections. (**D**) Representative images of the subgranular zone three weeks post EdU injection showing staining for EdU (red), DCX (magenta), CALB1 (green), and DAPI (blue). The arrowhead points to an EdU-labelled cell that is positive for DCX but negative for CALB1. The arrow points to an EdU-labelled cell that is double positive for DCX and CALB1. Scale bars = 20 µm. (**E**) Quantification of the percentage of EdU-labelled cells that were DCX^+^ in mice of the three genotypes. (**F**) Quantification of the relative proportion of EdU^+^ DCX^+^ cells that were CALB1^-^ or CALB1^+^ in mice of the three genotypes. *P* < 0.0001 using Chi-square test. N = 4–7 animals per group. In (**B**) and (**E**), data are presented in box-and-whisker plots showing all data points, where centre lines represent medians, box limits represent interquartile ranges, and whiskers represent minimum to maximum data ranges. Statistical analyses were performed with one-way ANOVA with Tukey’s post hoc test. *P < 0.05; **P < 0.01.

We next investigated the role of CIC outside of the precursor cell phase since we observed dynamic regulation of CIC during differentiation progression (Fig. 1). In the adult dentate gyrus, all newly generated neurons in the three survival and maturation phases express DCX^20–22^. DCX^+^ cells thus encompass cells before selective cell death and those that have survived and will persist for a long time. When we examined the DCX^+^ cell population in the control and *Emx1-Cre Cic* knockout mice, we did not find a difference in the total number of these cells among the three genotypes (Fig. 3A, B). This is somewhat surprising given that neural progenitor cells were reduced in the knockout mice (Fig. 2; Supplementary Fig. S5). In this context, an unchanged DCX^+^ cell population could be due to reduced cell death or altered differentiation progression. To investigate these possibilities, we first examined the number of DCX^+^ cells expressing the apoptosis marker cleaved-caspase 3 (CASP3). We did not detect any differences in cleaved CASP3^+^ cells among the three genotypes (Supplementary Fig. S6). However, it needs to be recognized that CASP3 activation is transient and that CASP3-independent programmed cell death pathways have been shown to regulate adult neurogenesis^23^. We next injected EdU into the mice (two injections that were two hours apart) and analysed them three weeks after injections (Fig. 3C). We chose this time point because this is when the EdU-labelled cells start to become mature neurons positive for calbindin (CALB1)^24–26^. We reasoned that this experimental paradigm would allow us to assess the effects of *Cic* deletion on the development of the DCX^+^ cells and their transition into CALB1^+^ mature neurons. We calculated the proportion of EdU-labelled DCX^+^ cells and found that the *Emx1-Cre Cic* knockout mice had a significantly higher percentage of these cells compared to the controls, indictive of increased cell survival and/or altered differentiation progression (Fig. 3D, E). Within the EdU-labelled DCX^+^ cell population, we observed CALB1 expression in ~ 25% of the cells in the control and heterozygous mice (Fig. 3D, F). Interestingly, we did not find any DCX and CALB1 co-expressing cells in the EdU-labelled population of the *Emx1-Cre Cic* knockout mice, suggesting impaired development of DCX^+^ immature neurons into CALB1^+^ mature neurons. Overall, our results strongly suggest that, in the *Emx1-Cre Cic* knockout mice, despite the reduction in the progenitor cell pool, the population of DCX^+^ cells remain similar in size with no obvious effect on cell death. However, loss of *Cic* increases progenitor differentiation into DCX^+^ cells and delays the transition of DCX^+^ cells into mature neurons.

### Loss of CIC leads to reduced dendritic arborization and abnormal migration of DCX^+^ cells

**Figure 4:**
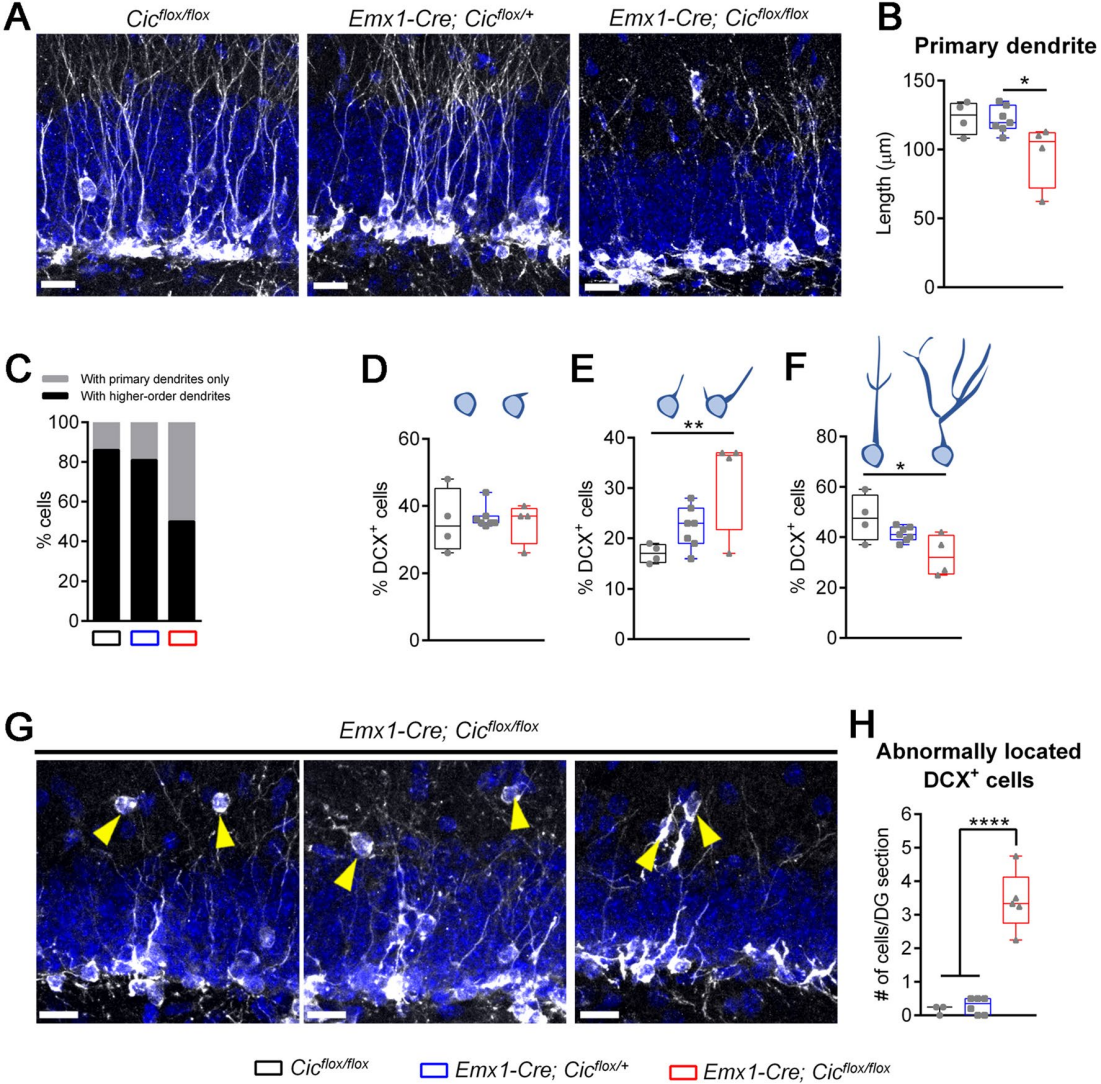
Reduced dendritic branching and abnormal migration of DCX^+^ cells in the *Emx1-Cre Cic* knockout mice. (**A**) Representative images of the dentate gyrus show abnormal dendritic morphology in knockout DCX^+^ cells. DAPI (blue) and DCX (grey). (**B**) Quantification of primary dendrite length. (**C**) Dendrite analysis shows reduced dendritic complexity in the knockout mice. *P* < 0.0001 using Chi-square test. (**D**–**F**) DCX^+^ cells are categorized into distinct subgroups based on their dendrite morphology. The *Emx1-Cre Cic* knockout mice show no difference in the number of the most immature subgroup with no or short processes (**D**), an increase in the intermediate subgroup with a medium-length process (**E**), and a decrease in the most mature subgroup with a thick long apical dendrite or an elaborate dendritic tree (**F**). (**G**) Representative images of the dentate gyrus show ectopically located DCX^+^ cells in the *Emx1-Cre Cic* knockout mice. Each image is from a different animal. DAPI (blue) and DCX (grey). (**H**) Quantification of abnormally located DCX^+^ cells. N = 3‒6 animals per group. Scale bars = 20 µm. Data are presented in box-and-whisker plots showing all data points, where centre lines represent medians, box limits represent interquartile ranges, and whiskers represent minimum to maximum data ranges. Statistical analyses were performed with one-way ANOVA with Tukey’s post hoc test. * *P* < 0.05; ** *P* < 0.01; ****P < 0.0001.

The period of DCX expression is associated with neuronal migration and neurite outgrowth: immature cells start expressing DCX when they begin to migrate and grow their dendrites, then they turn off DCX expression when they become fully mature^20–22^. As DCX expression spans the entire process of dendritic development, DCX^+^ cells exhibit varying degree of dendrite maturation. The least mature DCX^+^ cells have no or very short, plump processes. Then they develop an intermediate-length primary process perpendicular to the granular layer, followed by the establishment of a strong apical primary dendrite with elaborate dendritic branching in the molecular layer^22^. As such, the length of the primary dendrite and complexity of the dendritic architecture correlates with dendrite maturity of DCX^+^ cells. Our dendrite analysis showed that knockout DCX^+^ cells had shorter primary dendrites when compared to the control mice, indicating that they were less mature (Fig. 4A, B). When we focused on DCX^+^ cells with a primary dendrite, about 85% of these cells in the control and conditional heterozygous mice formed higher-order branching in the molecular layer. Only 15% of DCX^+^ cells in these mice had one single primary process lacking any secondary branches (Fig. 4C; Supplementary Fig. S7). In the knockout mice, however, only 50% of DCX^+^ cells had higher-order dendrites, and the rest of the 50% showed an immature morphology with a single intermediate-length primary dendrite that lacked any branches (Fig. 4C; Supplementary Fig. S7). These data demonstrate impaired dendrite development in the knockout mice.

To further analyze dendritogenesis in the *Emx1-Cre Cic* mice, we categorize DCX^+^ cells into three different developmental stages using a previously described morphology-based criteria: the most immature subgroup with no or short processes, the intermediate subgroup with a medium-length process, and the most mature subgroup with a strong primary process and elaborate branching^22^. While we found no difference in the number of DCX^+^ cells of the most immature subgroup (Fig. 4D), there was a significant increase in the intermediate subgroup (Fig. 4E) and a concomitant decrease in the most mature subgroup of DCX^+^ cells in the knockout mice (Fig. 4F). Altogether, these data demonstrate dendritic branching defects of DCX^+^ cells in the knockout mice, and pinpoint the exact dendrite development stage at which CIC plays a critical role.

We next asked whether loss of CIC had any effect on cell migration. During adult hippocampal neurogenesis, most migration occurs in DCX-expressing cell stages^3^. Radial migration of DCX^+^ cells is typically confined to the inner two thirds of the granule cell layer^3,27^. In agreement with this, we observed characteristic radial migration of DCX^+^ cells in the control and conditional heterozygous mice: most cells remained within the inner half of the granule cell layer and they seldomly migrate past the granule cell layer (Fig. 4A; Supplementary Fig. S7). In the knockout mice, while majority of DCX^+^ cells were seen in granule cell layer, we frequently and consistently found DCX^+^ cells in the molecular layer of the dentate gyrus (Fig. 4G, H; Supplementary Fig. S7). These abnormally located DCX^+^ cells lacked primary processes and extended their dendrites in a random fashion, features of a relatively immature phenotype. To further refine the molecular identity of these abnormally migrated DCX^+^ cells, we co-stained DCX with other neural cell type-specific markers. The cells were negative for the neural progenitor cell marker SOX2, the mature neuron marker NeuN, the astrocyte marker GFAP, and the oligodendroglial marker OLIG2, indicating that these were *bona fide* dentate gyrus immature cells (Supplementary Fig. S8). Indeed, we also observed some TBR2^+^ IPCs abnormally located to the molecular layer of the *Emx1-Cre Cic* knockout mice (Supplementary Fig. S5B), further demonstrating aberrant migration of immature neuronal lineage cells in the knockout mice.

In brief, CIC regulates dendritic development of immature neurons in the adult dentate gyrus. Loss of CIC impairs dendritic arborization of DCX^+^ cells, concomitant with uncontrolled radial migration of some of these cells.

### Increased NFIB expression is associated with abnormal development and migration of DCX^+^ cells in the *Emx1-Cre Cic* knockout mice

Adult hippocampal neurogenesis is driven by a coordinated network of transcription factors that orchestrate the developmental sequence of proliferation, differentiation and maturation^5^. As CIC regulates the expression of other transcription factors involved in cell growth and differentiation^28,29^, we hypothesized that deletion of CIC from the adult dentate gyrus disrupted downstream transcription factor networks. We reasoned that a transcription factor repressed by CIC would have an expression pattern inversely correlated with the expression of CIC along the neurogenic lineage. To identify such factors, we took a list of CIC-bound transcription factor genes in neural stem cells and looked for ones with low expression in neural progenitor cells and mature neurons but high expression in neuroblasts/immature neurons using a single-cell RNA sequencing dataset of the adult dentate gyrus^6,30^. This analysis revealed only two candidates, *Nfia* and *Nfib*, which are both members of the nuclear factor I (NFI) family (Supplementary Fig. S9 and S10).

By co-staining NFIA and NFIB with cell-stage specific markers, we confirmed that both NFIA and NFIB were most highly expressed in DCX^+^ cells, with NFIB experiencing a greater increase in expression from the transition of SOX2^+^ neural progenitor cells to DCX^+^ cells (Supplementary Fig. S9 and S10). Moreover, within the DCX^+^ population, strong NFIB expression was seen in majority of the cells except the ones with the most mature morphology, namely those with an elaborate dendritic tree, an enlarged cell body and low DCX expression^22,31,32^. As DCX^+^ cells developed into mature neurons, NFIA and NFIB levels dropped. Thus, expression of NFIA and NFIB along the granule cell lineage development inversely correlates with the expression of CIC (Supplementary Fig. S12).

**Figure 5:**
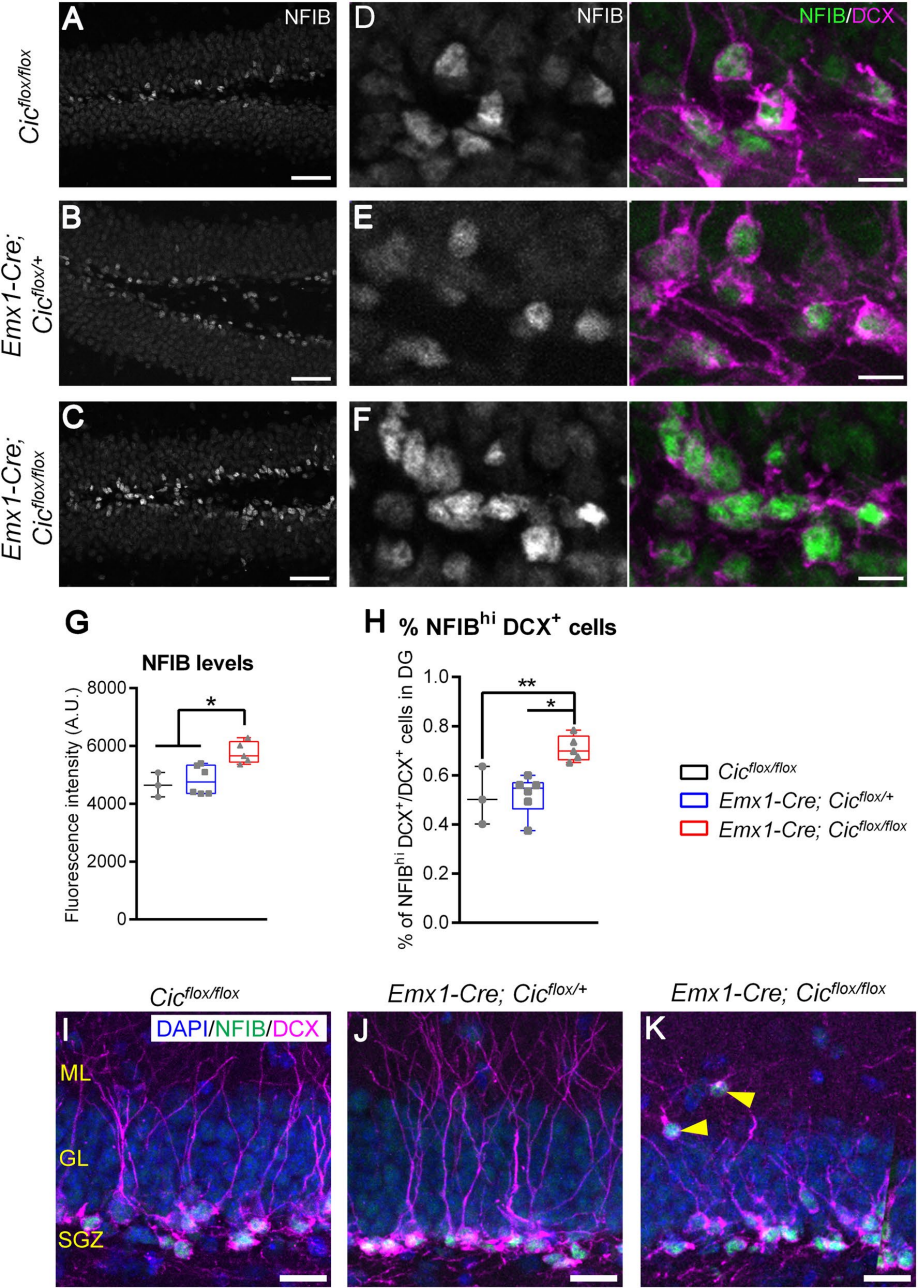
Upregulation of NFIB in neuroblasts of the *Emx1-Cre Cic* knockout mice. (**A**–**C**) Representative images of NFIB immunostaining (grey) in the dentate gyrus. Cells with the highest NFIB expression are found in the neurogenic subgranular zone. Scale bars = 50 µm. (**D**–**F**) Representative images of NFIB staining at higher magnification showing increased NFIB immunoreactivity and more NFIB^hi^ cells in the knockout mice. Scale bars = 10 µm. (**G**) Quantification of fluorescent staining intensity of NFIB in DCX^+^ cells. More than 150 cells per animal were analysed. Each data point represents the average value from multiple cells per animal. (**H**) Quantification of the percentage of DCX^+^ neuroblasts co-express high levels of NFIB. N = 3‒6 animals per group. (**I**–**K**) In the knockout mice, DCX^+^ cells abnormally located to the molecular layer express high levels of NFIB (yellow arrowheads). Scale bars = 20 µm. Data are presented in box-and-whisker plots showing all data points, where centre lines represent medians, box limits represent interquartile ranges, and whiskers represent minimum to maximum data ranges. Statistical analyses were performed with one-way ANOVA with Tukey’s post hoc test. *P < 0.05; **P < 0.01.

We next asked whether NFIA or NFIB expression was altered in the dentate gyrus of the *Emx1-Cre Cic* knockout mice. While deletion of CIC did not lead to ectopic NFIA or NFIB expression in neural progenitor cells or mature neurons, NFIB (but not NFIA) levels were significantly upregulated in DCX^+^ cells of the *Emx1-Cre Cic* knockout mice compared with the control mice (Fig. 5A–G), supporting the idea that CIC regulates NFIB expression in DCX^+^ cells. As downregulation of NFIB is associated with neuronal maturation (Supplementary Fig. S10), we then asked whether increased levels of NFIB in the *Emx1-Cre Cic* knockout mice would alter the development of DCX^+^ cells. In the control mice, about 50% of DCX^+^ cells expressed NFIB at levels higher than those in SOX2^+^ progenitor cells; these NFIB^hi^ DCX^+^ cells exhibited a relatively immature morphology compared to DCX^+^ cells with low NFIB expression. In the knockout mice, however, roughly 75% of DCX^+^ cells co-expressed high levels of NFIB (Fig. 5C, F, H). Given that high NFIB expression in DCX^+^ cells molecularly defines a less mature phenotype (Supplementary Fig. S10), this result indicates an expansion of the less mature DCX^+^ subgroups in the knockout mice. Since the total number of DCX^+^ cells did not differ between the different lines of mice (Fig. 3B), this result further points to a reduction in the most mature DCX^+^ subgroup in the knockout mice. Interestingly, all *Cic*-knockout DCX^+^ cells that had abnormally migrated into the molecular layer had strong NFIB expression (Fig. 5I–K), raising the possibility that aberrant NFIB levels might contribute to abnormal neuronal migration. Altogether, these data suggest that deletion of CIC from the adult hippocampus leads to upregulation of NFIB in DCX^+^ cells, concomitant with an expansion of the less mature subgroups of these cells and occasional abnormal migration.

## Discussion

In the present study, we demonstrate that CIC is dynamically regulated during neuronal lineage progression in the adult dentate gyrus. Using the *Emx1-Cre Cic* knockout mice, we have made three key observations. (1) Deletion of CIC from the dentate gyrus reduces the adult progenitor cell pool without affecting the proliferation potential of progenitor cells. (2) Despite the reduction in the progenitor cell pool, *Cic* deletion does not alter the number of DCX^+^ cells. This is likely due to altered differentiation progression, as we did not find an apparent effect on cell death of the DCX^+^ population. (3) Loss of CIC impedes dendritic development and migration of maturing neurons. Our findings provide evidence for an important role of CIC in adult hippocampal neurogenesis.

CIC is a transcriptional repressor that recruits other co-repressor proteins, such as the mSWI/SNF and SIN3-HDAC complexes, to repress target gene expression^11,30^. It is widely expressed in the embryonic and adult brains^12^. Within neurogenic regions, CIC expression is low in neural stem and progenitor cells but high in fully differentiated neurons^11,33^. However, whether CIC upregulation during neurogenesis is strictly linear or has additional dynamics is unknown. Here, by comparing CIC expression in different cell stages, we made a surprising discovery that CIC is first downregulated in DCX^+^ cells and then upregulated as new neurons exit the DCX-expressing stage. The dip in CIC levels in DCX^+^ cells may allow for the onset of a transcriptional program that is uniquely required for the differentiation and/or survival of these cells. This notion is supported by the results of our EdU-labelling study showing increased percentage of EdU^+^ DCX^+^ cells in the knockout mice, which suggests increased DCX^+^ cell survival and/or increased progenitor cell differentiation into DCX^+^ cells upon the loss of CIC. New neurons begin to express DCX when they start to migrate and grow their dendrites^20,22^. These specialized behaviours must be supported by a set of genes that promote cell motility and proper neurite outgrowth. CIC may regulate some of these genes as we observed impaired dendritic development and migration of DCX^+^ cells when we deleted CIC from the dentate gyrus. What mediates the dynamic expression of CIC during neurogenesis remains to be investigated, but CIC is known to be negatively regulated by the growth factor–RAS/MAPK signalling pathway^28,29^. Therefore, CIC downregulation in DCX^+^ cells may serve to integrate external neurogenic cues, such as growth factor signalling, to neuronal lineage development by enabling appropriate activation of genes involved in survival and/or neuronal differentiation, cell motility and dendritic outgrowth.

As a gatekeeper of the RAS/MAPK pathway, CIC restricts cell proliferation in the contexts of development and cancer^10^. Surprisingly, we did not find a change in the proliferation potential of SOX2^+^ progenitor cells when CIC was deleted from the dentate gyrus. Our observation is in agreement with a previous study using the *Emx1-Cre Cic* knockout mice to demonstrate that loss of CIC does not alter the proliferation rate of embryonic ventricular zone neural progenitor cells^12^. On the other hand, deletion of CIC increases proliferation of cultured neural progenitor cells isolated from the embryonic ventricular zone and perinatal subventricular zone^11,33^. Such discrepancy may arise from the use of in vivo versus in vitro systems. It is possible that when taken out of the neurogenic microenvironment and cultured in vitro, *Cic*-null neural stem cells sustain cell proliferation via a cell-autonomous mechanism. In vivo, however, proliferation of progenitor cells is regulated by a plethora of extracellular signals in the neurogenic niche^2,5^. Diverse regulatory feedback signals might keep the proliferation capacity of *Cic* knockout progenitor cells in check to prevent precocious stem cell pool exhaustion. Although the proliferation potential of neural progenitor cells is not changed, the progenitor cell pool is reduced in the *Emx1-Cre Cic* knockout mice. This could be due to increased progenitor cell differentiation into DCX^+^ cells as our data suggest. Alternatively, there might be a defect in the initial establishment of adult neural progenitors in the knockout dentate gyrus. Future studies are needed to determine whether CIC plays a role in the early postnatal dentate gyrus to establish adult neural progenitors.

A striking phenotype uncovered in the absence of CIC is the disruption of DCX^+^ cell development. This is supported by (1) EdU-labelling study showing impaired progression of knockout DCX^+^ cells into CALB1^+^ mature neurons, (2) morphological analysis revealing reduced dendritic complexity in knockout DCX^+^ cells, and (3) molecular analysis demonstrating an expansion of DCX^+^ cells co-expressing high levels of NFIB, which marks the less mature subgroups. Previous studies have demonstrated a role for CIC in dendrite development and neuronal maturation during early brain development. Deletion of CIC reduces dendritic arborization of layers 2–4 neurons in the cerebral cortex during early postnatal development^12^. Therefore, CIC may regulate dendritic development across different neuronal subtypes. Moreover, deletion of CIC using the pan-neural *Nestin-Cre* driver expands the pool of neuroblasts with a less mature phenotype in the early postnatal (2-week-old) dentate gyrus^11^. CIC thus seems to play a consistent role in dentate gyrus neurogenesis whether in early postnatal or adult periods. In addition to highlighting the requirement of CIC for dendritic development of DCX^+^ cells, our data suggest that CIC also plays a role in the regulation of migration of these cells. DCX + cells normally do not migrate pass the granule cell layer^3,27^. However, we consistently observed DCX^+^ cells abnormally located in the molecular layer of the *Emx1-Cre Cic* knockout mice. Notably, only a small fraction of DCX^+^ cells in the *Cic* knockout mice exhibit abnormal migration. This might be due to other redundant/compensatory pathways that safeguard global neuronal migration in the absence of CIC. As little is known about the molecular mechanisms underlying neuronal migration in the adult dentate gyrus, future studies that aim to capture these abnormally migrated DCX^+^ cells and interrogate their molecular signatures using single-cell approaches may yield new insight into the regulation of migration of adult-born neurons.

The nuclear factor I (NFI) family of transcription factors are important players in brain development. Our results demonstrate that within the adult hippocampal neurogenic lineage, NFIA and NFIB both have peak expression at the DCX^+^ cell stage, a pattern similar to that of NFIX^13^. While NFIX has been shown to be required for neuroblast maturation and dendritic development, the role of NFIA and NFIB in the adult dentate gyrus needs to be investigated. Much of our knowledge of the NFI factors in normal brain development comes from loss-of-function studies^34^. The consequences of ectopic or overexpression of these factors remain largely unexplored. Nonetheless, evidence suggests that the brain is highly sensitive to the dosage of NFI factors. Astrocyte differentiation is reduced and delayed as a result of NFIB loss, while precocious astrogliosis is observed upon ectopic expression of NFIB^35–37^. Adult hippocampal neurogenesis is biased toward an oligodendrocyte cell fate by NFIX deletion or NFIB overexpression in neural stem cells^13,38^. Importantly, inhibition of NFI factors in developing cerebellar granule neurons impedes neuronal process formation and migration via downregulation of cell adhesion molecules^39^. Our finding that upregulation of NFIB in CIC knockout DCX^+^ cells is associated with abnormal dendritic development and migration further attests to the importance of NFI factors in neuronal maturation, and raises the possibility that proper neuronal migration and maturation requires an optimal dosage of NFI factors. Overexpression of NFI factors might lead to increased expression of cell adhesion molecules, disrupting the normal migratory behaviour of DCX^+^ cells. Interestingly, we observe upregulation only of NFIB and not NFIA in the *Emx1-Cre Cic* knockout mice, suggesting differential regulation of the two factors by CIC or compensatory mechanisms that maintain the steady-state levels of NFIA in the absence of CIC. The NFI factors have very similar expression patterns during brain development and overlapping transcriptional targets^40,41^, but how they might be regulated differently in diverse cellular contexts is not well understood. Our results open the door for future investigative studies to dissect the mechanisms by which NFI factors are differentially modulated in the adult neurogenic lineage. Moreover, it is intriguing that loss of CIC only increased NFIB levels in DCX^+^ cells, where CIC is normally kept at a very low level. Future studies are required to test if CIC directly regulates *Nifb* and whether upregulation of NFIB is a cause or consequence of altered development of DCX^+^ cells.

Although our present data reveal several abnormalities of adult hippocampal neurogenesis in the *Emx1-Cre Cic* knockout mice, it needs to be recognized that the observed defects may partly arise from *Cic* deletion in the developing brain, as the cre recombinase is turned on in forebrain progenitors from E9.5^14^. However, it is worth noting that the *Emx1-Cre Cic* knockout mice have a grossly normal dentate gyrus with similar cellularity to the control mice at 5 weeks of age^12^. We also did not find a significant difference in the size of the neurogenic SGZ in 11-week-old mice. Nonetheless, loss of *Cic* in mature granule neurons in the *Emx1-Cre Cic* knockout mice may change the local environment of adult neurogenesis, contributing to the observed defects in a non-cell autonomous manner. Therefore, establishing a mechanistic or a causative link will required further studies using knockout mice with conditional *Cic* inactivation in adult neural stem cells (such as *Cic* deletion using the *Nes-Cre/ERT2* driver line^42^).

In conclusion, our present study identifies CIC as a critical regulator of adult dentate gyrus neurogenesis and highlights the requirement of CIC for proper dendritic development of DCX^+^ cells (Supplementary Fig. S12). The highly branched dendrites of DCX^+^ immature neurons provide the anatomical basis for receiving information from the entorhinal cortex, enabling ongoing learning in the adult brain^43,44^. Therefore, dendritic arborization defects in these cells could contribute to the previously observed learning and memory deficits in the *Emx1-Cre Cic* knockout mice^12^. Moreover, heterozygous loss-of-function mutations in *CIC* cause a rare neurodevelopmental syndrome characterized by prominent intellectual disability/learning difficulties in affected individuals^12,45^. Given that these pathologies persist into adulthood, our finding that CIC is required for adult hippocampal neurogenesis sets the stage for future investigative studies to understand whether the loss of *CIC* triggers ongoing learning difficulties in adult patients by impairing adult neurogenesis. Beyond the *CIC* haploinsufficiency syndrome, CIC may also play a broader role in age-related cognitive decline, as impaired adult hippocampal neurogenesis is believed to contribute to age-associated cognitive deficits^46–48^. Aging leads to a reduction in neural progenitor cells^49–51^ and a delay in morphological maturation of adult-born granule neurons^52^. Both of these defects were observed in the *Emx1-Cre Cic* knockout mice. In future studies, it will be interesting to determine whether CIC expression along the adult neurogenic lineage is altered by aging, and whether aging exacerbates the neurogenesis defects of the *Emx1-Cre Cic* knockout mice.

## Materials and methods

### Mouse model

Generation of the *Cic*^*flox*^ mice has been previously described^12^. The *Cic*^*flox*^ mice are also available from The Jackson Laboratory (stock number: 030555). Wildtype C57BL/6J mice and *Emx1-Cre* mice [B6.129S2-*Emx1*^*tm1(cre)Krj*^/J, stock number: 005628] were obtained from The Jackson Laboratory. No sex differences were observed in our studies. Both male and female mice were used for experiments. For studies involving wildtype C57BL/6J animals, animals were eight weeks of age. For EdU labelling experiments, 8-week-old mice were used. For all other experiments, 11- to 20-week old *Emx1-Cre; Cic*^*flox/flox*^ mice were used, and their littermate *Cic*^*flox/flox*^ and *Emx1-Cre; Cic*^*flox/*+^ mice were used as controls.

### Ethics statement

All procedures in mice were approved by the Animal Care and Use Committee of the University of Alberta. All methods were performed in accordance with the relevant guidelines and regulations. The study was carried out in compliance with the ARRIVE guidelines: https://arriveguidelines.org.

### 5-Ethynyl-2′-deoxyuridine (EdU) labelling experiment in mice

EdU (Santa Cruz, sc-284628) was reconstituted in DMSO to a concentration of 100 mg/mL, aliquoted and stored at − 20 °C until further use. For progenitor proliferation analysis, on the day of experiment, EdU was diluted with endotoxin-free PBS to a final concentration of 5 mg/ml, and injected intraperitoneally into 8-week-old mice to a final in vivo concentration of 50 µg/g body weight. A second dose of EdU was injected two hours after the first injection to maximize labelling efficiency. Mice were euthanized two hours or three weeks after the second injection by an intraperitoneal injection of sodium pentobarbital and used for brain section preparation.

### Preparation of mouse brain sections

Mice were deeply anaesthetized via intraperitoneal injection of sodium pentobarbital, and transcardially perfused first with PBS then with 4% paraformaldehyde in PBS. Mouse brains were then dissected and immersed in a 4% paraformaldehyde solution overnight at 4 °C to further fix the tissues, followed by submerging the brains in cryoprotective 15% and 30% sucrose, each for 24 h. The mouse brains were cut coronally using a brain matrix and cryo-embedded using Tissue-Tek OCT compound. Coronal brain sections (40 μm-thick) were cut using a cryostat (Leica Microsystems Inc., Buffalo Grove, IL, USA), transferred to ColorFrost Plus Microscope Slides (Fisher Scientific, 22-230-890), and air-dried for immunofluorescence staining. Excess slides were stored at − 80 °C. Frozen slides were thawed at room temperature and dried for several hours to overnight before undergoing staining procedures.

### EdU labelling detection

EdU detection was performed by using the Click-iT Alexa Fluor 555 dye EdU kit (Invitrogen, C10338) according to the manufacturer’s protocol. Briefly, slides with brain sections were post-fixed with 4% paraformaldehyde, washed with 3% bovine serum albumin in PBS, then permeabilized with 0.5% Triton-X 100 in PBS at room temperature for 20 min. Slides were then incubated with the Click-iT reaction cocktail that contained Click-iT reaction buffer, Alexa Fluor 555 Azide, CuSO4, and reaction buffer additive for 30 min while being protected from light. Slides were then washed once with 3% BSA in PBS and then three times with PBS before being further processed for immunofluorescence staining or counterstaining with DAPI.

### Immunofluorescence staining

Immunofluorescence staining was performed as previously described with modifications^12^. Slides containing brain sections were first post-fixed in 10% neutral buffered formalin (Fisher Scientific, SF98-4) for 5–10 min, then washed three times with PBS. Antigen retrieval was performed using the Antigen Unmasking Solution (Vector Laboratories, H-3300) at 95 °C for 20–60 min in a water bath with gentle shaking (60 rpm). Slides were cooled to room temperature and permeabilized with 0.3% Triton X-100 in PBS, then blocked with 5% normal donkey serum in 0.3% Triton X-100 in PBS. Slides were incubated at 4 °C overnight with the primary antibodies in blocking buffer. The slides were washed three times with 0.3% Triton X-100 in PBS, then incubated with secondary antibodies in blocking buffer at room temperature for two hours. The slides were then washed twice with 0.3% Triton X-100 in PBS, and then once with PBS. Autofluorescence quenching was carried out to reduce background autofluorescence using the Vector TrueVIEW Autofluorescence Quenching Kit (Vector Laboratories, SP-8400) and all slides within one experiment were treated similarly. Slides were counterstained with DAPI and then mounted using VECTASHIELD Vibrance mounting media (Vector Laboratories, H-1700).

### Antibodies

The following primary antibodies were used for immunofluorescence staining: rabbit anti-Ki67 (Abcam, ab15580, 1:1000), rabbit anti-CIC (Lu et al. 2017; 1:500), mouse anti-DCX (Santa Cruz Biotech, sc-217190; 1:25), goat anti-SOX2 (R&D Systems, AF2018; 1:500), mouse anti-NES (Abcam, ab11306; 1:200), rabbit anti-TBR2 (Abcam, 183,991; 1:500), rabbit anti CALB1 (Swant, CB38; 1:500), rabbit anti-NeuN (Abcam, ab177487; 1:500), rabbit anti-GFAP (DAKO, Z0334; 1:2000), rabbit anti-OLIG2 (Millipore, AB9610; 1:1000), rabbit anti-NFIB (Invitrogen, PIPA552032; 1:200), and rabbit anti-NFIA (Sigma, HPA006111-100UL; 1:200). The secondary antibodies used were donkey anti-rabbit Alexa Fluor 488 (Invitrogen, A21206; 1:1000), donkey anti-mouse Alexa Fluor 647 (Invitrogen, A31571; 1:1000), and donkey anti-goat Alexa Fluor 555 (Invitrogen, A21432; 1:1000).

### Confocal microscopy and image and data analyses

Immunofluorescent images were taken using a laser-scanning confocal microscope (Zeiss LSM 700). For each animal, tiled and z-stacked images of the dentate gyrus were acquired from at least three comparable coronal sections spanning the entire dorsal dentate gyrus and analyses were performed on images of these sections. Each data point represents the average value from multiple sections per animal. Quantifications of fluorescence intensity and cell counts were carried out using ImageJ. Cell counts for total SOX2^+^ and SOX2^+^ Ki67^+^ cells were restricted to cells within the subgranular zone of the dentate gyrus, which was defined as “a layer of cells expanding 5 μm into the hilus and 15 μm into the granular cell layer” as previously described^15^. Dendrite analyses were carried out using the Simple Neurite Tracer plugin of ImageJ. To quantify NFIA and NFIB immunofluorescence intensity and the number of cells expressing high levels of these proteins, images of single fluorescence channel were adjusted using the MaxEntropy method and an automated threshold calculated by ImageJ. The images were further subjected to Watershed binary processing to split cell clusters into individual cells. Particle analyses were then performed on the processed images, which yielded the number of cells expressing NFIA or NFIB and the mean fluorescence intensity of these cells. Statistical analyses were performed using GraphPad Prism. Detailed information on statistical analyses is given in the figure legends.

## Supplementary Information

Supplementary Information.

## Data Availability

All data generated or analysed during this study are included in this published article (and its Supplementary Information file).
