# Supplementary material for: Capicua regulates the development of adult-born neurons in the hippocampus
Source: Sci Rep. 2021 Jun 3;11:11725. doi: 10.1038/s41598-021-91168-5 (PMC8175746; doi:10.1038/s41598-021-91168-5)
Supplement: Supplementary file 1 — Supplementary Information. [file 41598_2021_91168_MOESM1_ESM.docx]

***Supplementary Information***

**Capicua regulates the development of adult-born neurons in the hippocampus**

Brenna Hourigan^1#^, Spencer D. Balay^1#†^, Graydon Yee^1^, Saloni Sharma^1^, Qiumin Tan^1*^

^1^Department of Cell Biology, University of Alberta, Edmonton, Canada T6J 2H7

^#^These authors contributed equally.

^†^Present address: Research Institute of Molecular Pathology, Vienna Biocenter, Campus-Vienna-Biocenter 1, 1030 Vienna, Austria.

^*^Corresponding author: qiumin@ualberta.ca

**Figure S1**

**
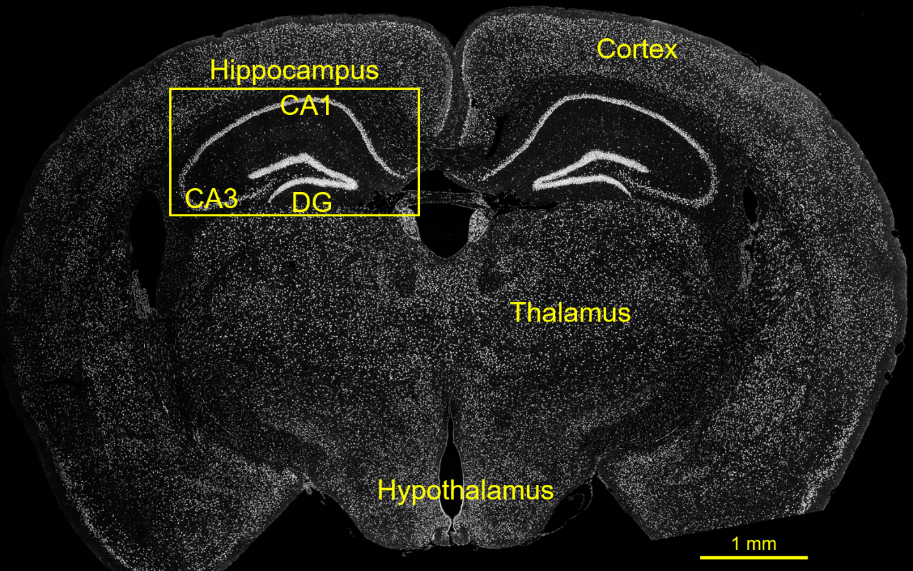
**

**Figure S1. CIC expression in the adult mouse brain**. Wildtype mouse brains were immunostained for CIC (grey). CIC is expressed most strongly in the dentate gyrus (DG) of the hippocampus. Scale bar = 1 mm.

**Figure S2**

**
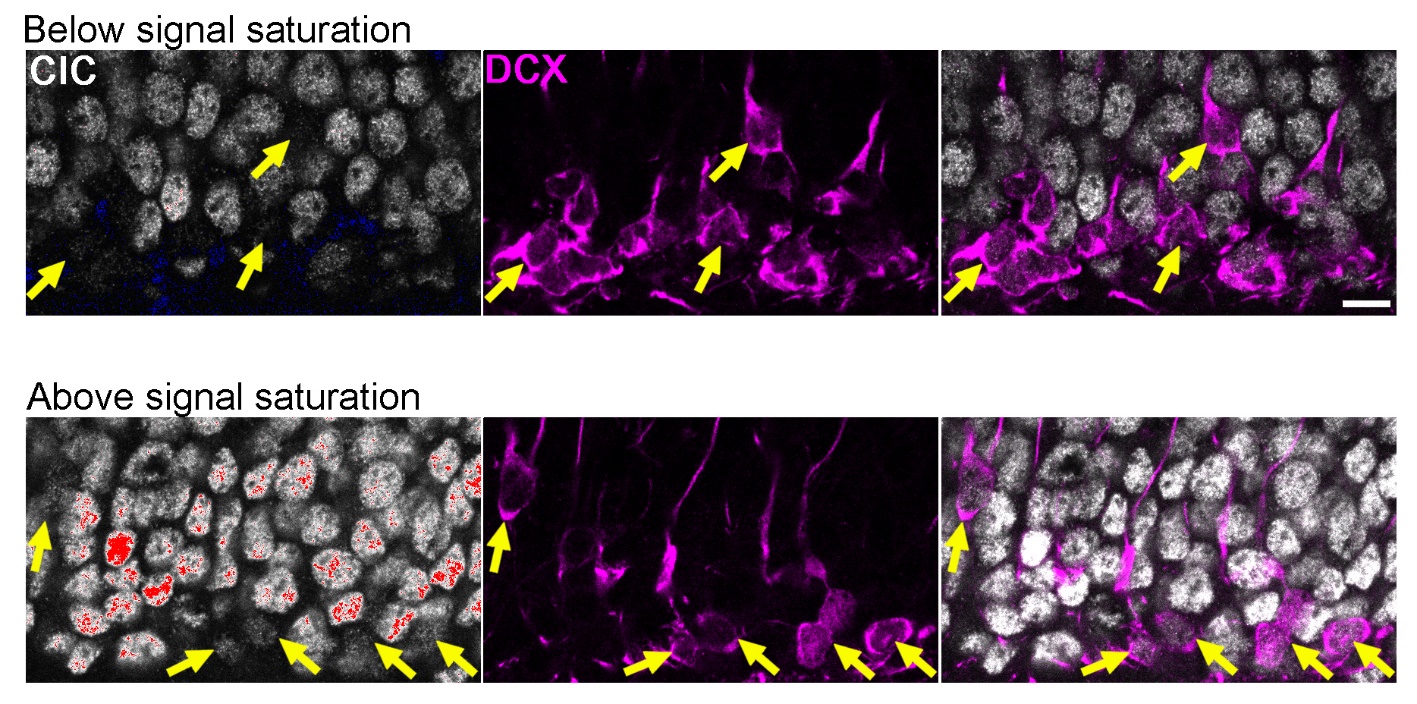
**

**Figure S2. CIC is expressed at a low but detectable level in DCX^+^ cells. *Upper panel***, same images as in Fig. 1C. To quantify the expression levels of CIC in multiple cell stages and to keep the fluorescence signals within the dynamic range, these images were taken below signal saturation in mature neurons using relatively low laser power and detector gain (saturated pixels in red; few pixels in the image are saturated). As a result, CIC appears to be not expressed in DCX^+^ cells (arrows). However, as shown in the ***lower panel***, when images were taken above signal saturation using high laser power and detector gain (saturated pixels in red), CIC is clearly visible in DCX^+^ cells (arrows). Scale bar = 10 µm.

**Figure S3**

**
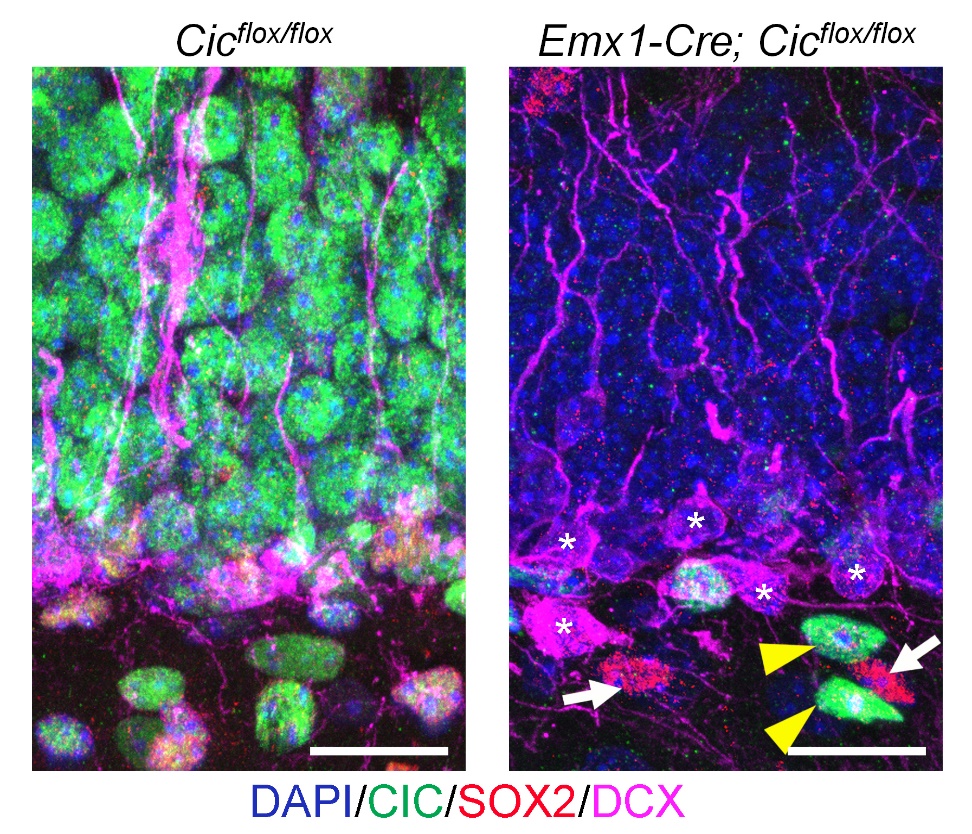
**

**Figure S3. CIC is efficiently deleted from the adult dentate gyrus of *Emx1-Cre* *Cic* knockout mice.** Representative confocal images of control (*Cic^flox/flox^*) and knockout (*Emx1-Cre*; *Cic^flox/flox^*) mice stained for DAPI (blue), CIC (green), SOX2 (red), and DCX (magenta). In the knockout mice, CIC is efficiently deleted from SOX2^+^ neural progenitor cells (white arrows), DCX^+^ neuroblasts (white asterisks), and mature neurons, which are defined as cells in the granular layer. Remaining CIC-expressing cells (yellow arrowheads) in the knockout mice are likely non-*Emx1*-lineage cells such as inhibitory neurons and microglia. Scale bars = 20 µm.

**Figure S4**

**
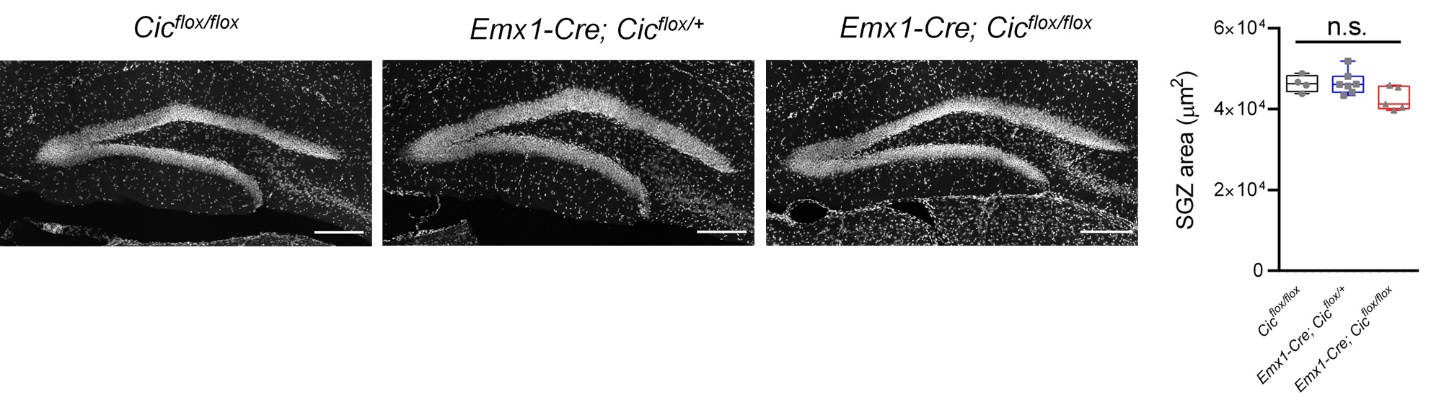
**

**Figure S4. The size of the adult subgranular zone is not significantly altered in the *Emx1-Cre Cic* knockout mice.** Representative confocal images of 11-week-old mice stained for DAPI (gray) to show the overall morphology of the adult dentate gyrus. Quantification of the areas of the subgranular zone (SGZ) is shown to the right. SGZ is defined as “a layer of cells expanding 5 μm into the hilus and 15 μm into the granular cell layer” as previously described (Sierra et al. 2010 *Cell Stem Cell*). N = 4–7 animals. Data are presented in box-and-whisker plots showing all data points, where centre lines represent medians, box limits represent interquartile ranges, and whiskers represent minimum to maximum data ranges. Statistical analysis was performed with one-way ANOVA with Tukey’s *post hoc* test. n.s., not significant.

**Figure S5**

**
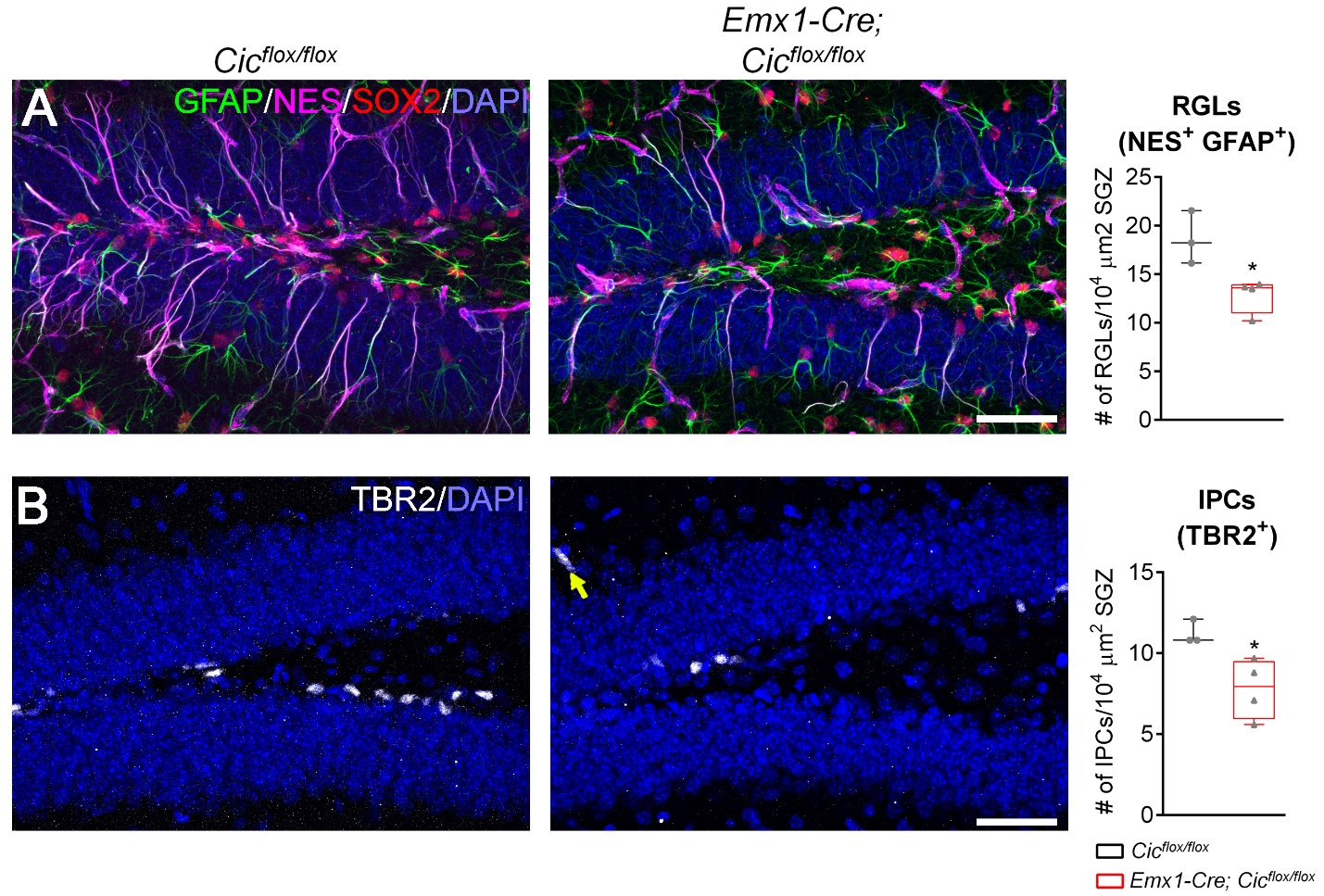
**

**Figure S5. Adult neural progenitor cells are reduced in the dentate gyrus of the *Emx1-Cre* *Cic* knockout mice.** (**A**) The *Emx1-Cre* *Cic* knockout mice have fewer GFAP^+^ NES^+^ radial glia-like (RGL) cells. Note that GFAP also marks astrocytes, and NES also marks pericytes of the vasculature, but only RGL cells are double positive for GFAP and NES. Quantification is shown to the right. (**B**) The *Emx1-Cre* *Cic* knockout mice have fewer TBR2^+^ intermediate progenitor cells (IPCs). An abnormally located TBR2^+^ IPC in the molecular layer is indicated by an arrow. Scale bars = 50 µm. N = 3‒4 animals per group. Data are presented in box-and-whisker plots showing all data points, where centre lines represent medians, box limits represent interquartile ranges, and whiskers represent minimum to maximum data ranges. Statistical analysis was performed with one-way ANOVA with Tukey’s *post hoc* test. *, *P* < 0.05.

**Figure S6**

**
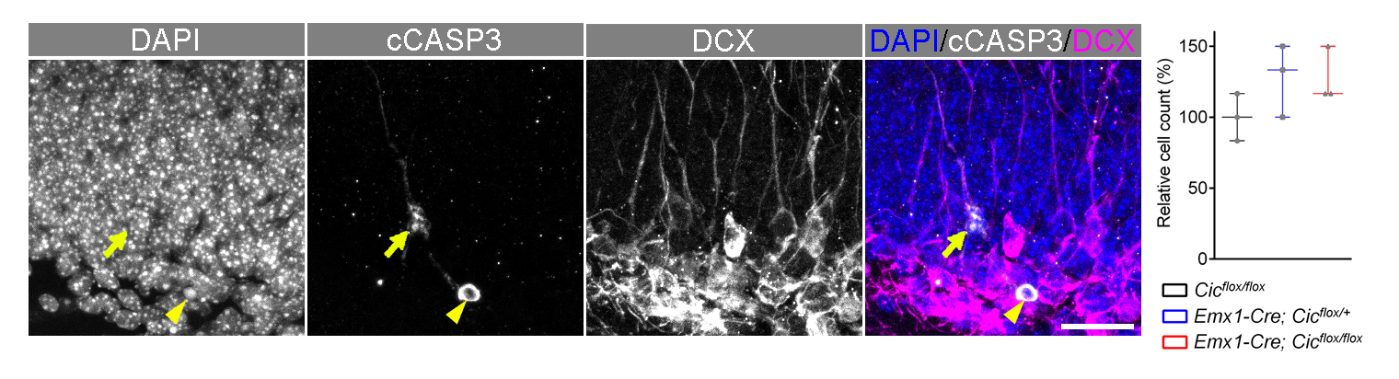
**

**Figure S6. Apoptotic cell death of DCX^+^ cells is unaltered in the *Emx1-Cre* *Cic* knockout mice.** Immunostaining for the apoptosis marker cleaved-caspase 3 (cCASP3). Quantification is shown to the right. Scale bars = 25 µm. N = 3 animals per group. Data are presented in box-and-whisker plots showing all data points, where centre lines represent medians, box limits represent interquartile ranges, and whiskers represent minimum to maximum data ranges. Statistical analysis was performed with one-way ANOVA with Tukey’s *post hoc* test.

**Figure S7**

**
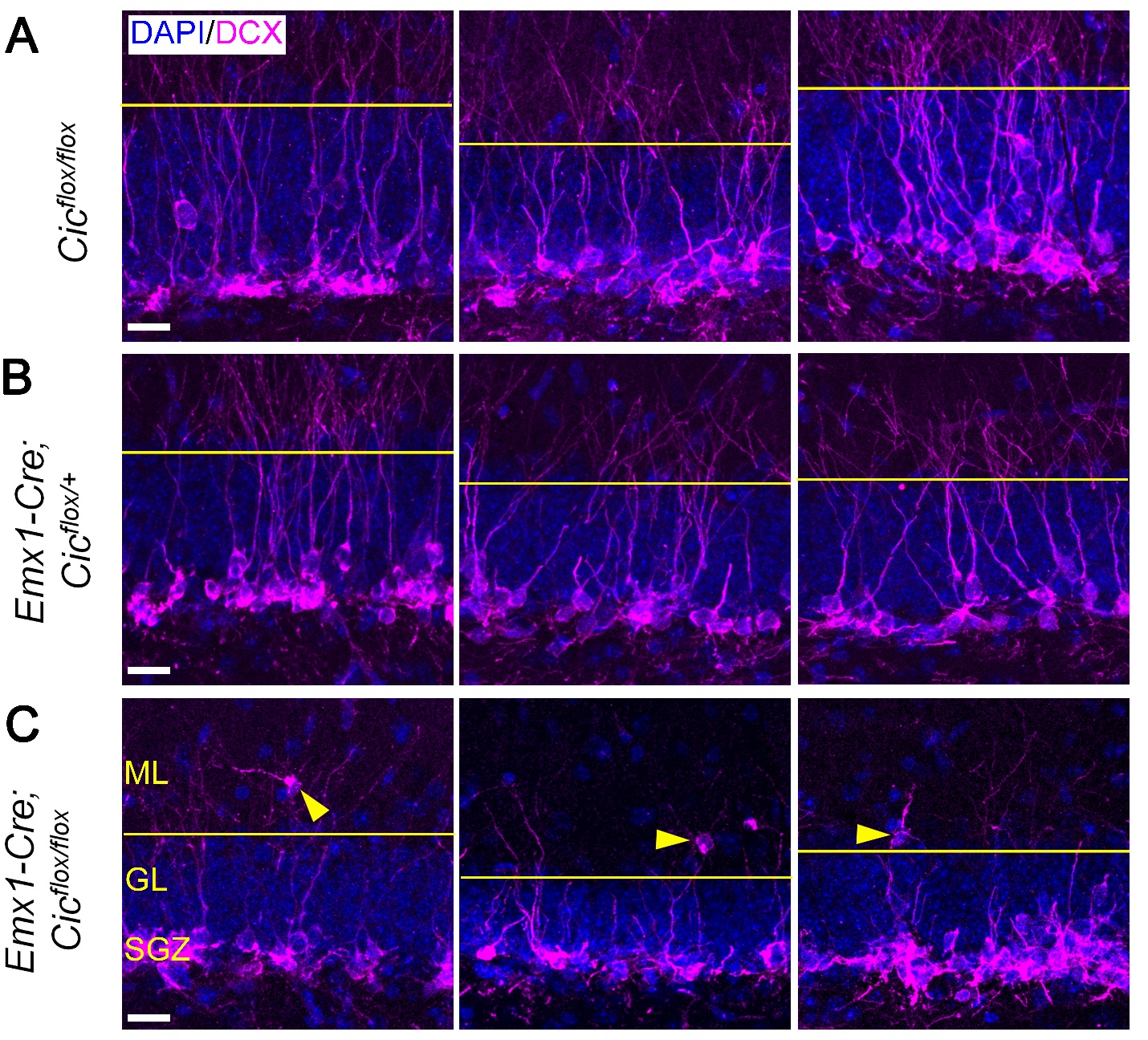
**

**Figure S7. DCX^+^ cells in *Emx1-Cre* *Cic* knockout mice show morphological and migration defects.** Representative images of the dentate gyrus in (A) control, (B) heterozygous, and (C) knockout mice, with DAPI staining (blue) and DCX (magenta). The yellow lines mark the boundary of granule cell layer (GL) and molecular layer (ML). Majority of DCX^+^ cells in the control (**A**) and conditional heterozygous (**B**) mice have similar morphology: they project a single apical dendritic process radially through the granule cell layer; these processes then branch to form dendritic arbours in the molecular layer. In contrast, most DCX^+^ cells in the knockout mice (**C**) fail to extend an apical dendritic process and instead produced random, non-radial outgrowths. DCX^+^ cells normally do not migrate into the molecular layer. However, DCX^+^ cells are frequently found in the molecular layer of the knockout mice (yellow arrowheads). These abnormalities were found in all knockout animals examined (N = 6). Representative images from three different animals from each genotype are shown. SGZ, subgranular zone. Scale bars = 20 µm.

**Figure S8**

**
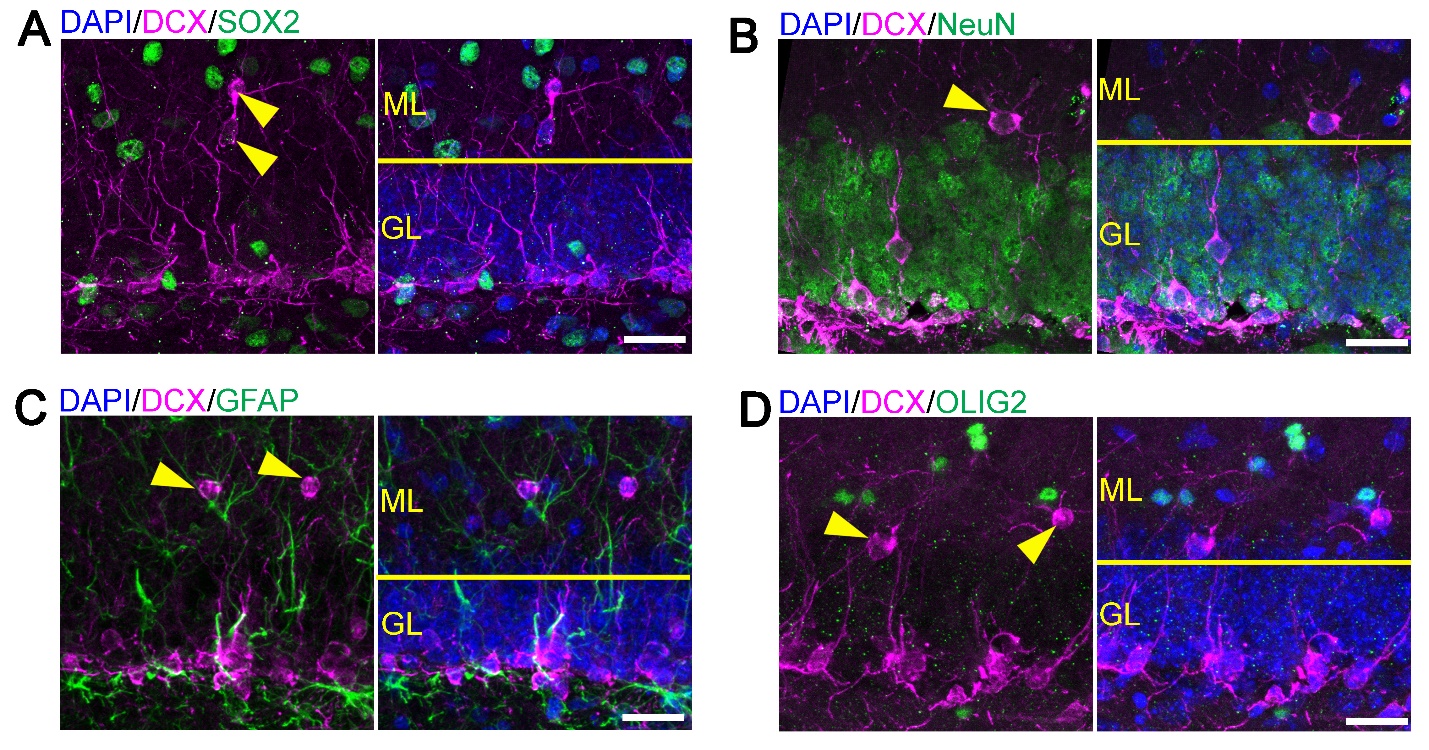
**

**Figure S8. Molecular characteristics of abnormally migrated DCX^+^ cells in the *Emx1-Cre* *Cic* knockout mice.** Representative images of the dentate gyrus in the *Emx1-Cre* *Cic* knockout mice show that DCX-expressing cells that have abnormally migrated into the molecular layer (yellow arrowheads) are negative for the neural progenitor cell marker SOX2 (**A**), the mature neuron marker NeuN (**B**), the astrocyte marker GFAP (**C**) and the oligodendroglial marker OLIG2 (**D**). Yellow lines mark the boundary between the granule cell layer (GL) and the molecular layer (ML). Scale bars = 25 µm.

**Figure S9**

**
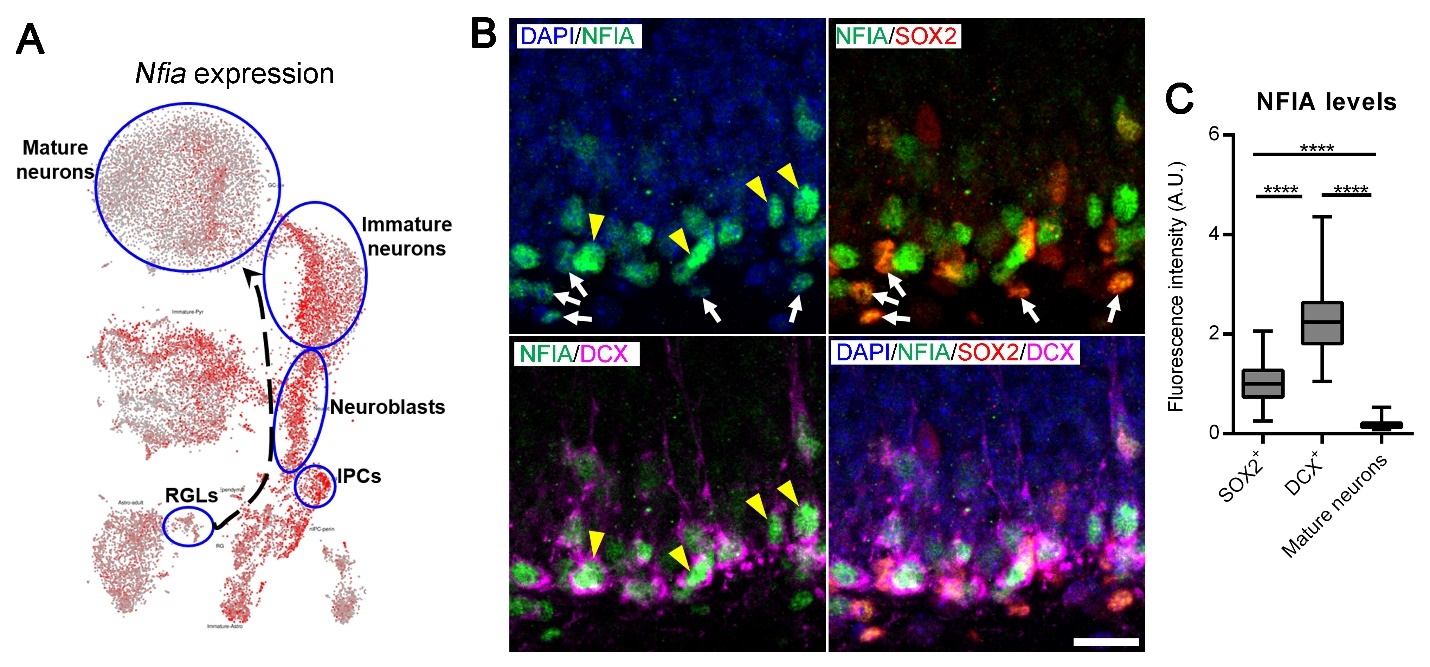
**

**Figure S9. Expression pattern of NFIA during adult hippocampal neurogenesis.** (**A**) tSNE plot of adult dentate gyrus single-cell RNA sequencing data showing *Nfia* gene expression along the developmental trajectory. Highest mRNA levels of *Nfia* are found in intermediate progenitor cells (IPCs), neuroblasts, and immature neurons. RGLs, radial glial like cells. *Nfia* levels drop when neurons become mature. The data is generated using an online browser from the Linnarsson lab (http://linnarssonlab.org/dentate/). (**B**) Representative images of NFIA and cell stage-specific marker expression in the dentate gyrus of adult control mice, showing DAPI (blue), NFIA (green), SOX2 (red) and DCX (magenta) staining. During adult hippocampal neurogenesis, peak NFIA levels are found in DCX^+^ cells (yellow arrowheads). NFIA is not detectable in mature granule neurons. White arrows point to SOX2^+^ neural progenitor cells, in which NFIA shows moderate expression. The expression pattern of NFIA protein is similar to its mRNA expression shown in (A). Scale bar = 20 µm. (**C**) Quantification of relative fluorescence intensity of NFIA immunostaining in different cell stages. N = 99 SOX2^+^ cells, 109 DCX^+^ cells, and 41 mature neurons from three 11-week-old control mice. Data are presented in box-and-whisker plots, where centre lines represent medians, box limits represent interquartile ranges, and whiskers represent minimum to maximum data ranges. Statistical analysis was performed with one-way ANOVA with Tukey’s *post hoc* test. **** *P* < 0.0001.

**Figure S10**

**
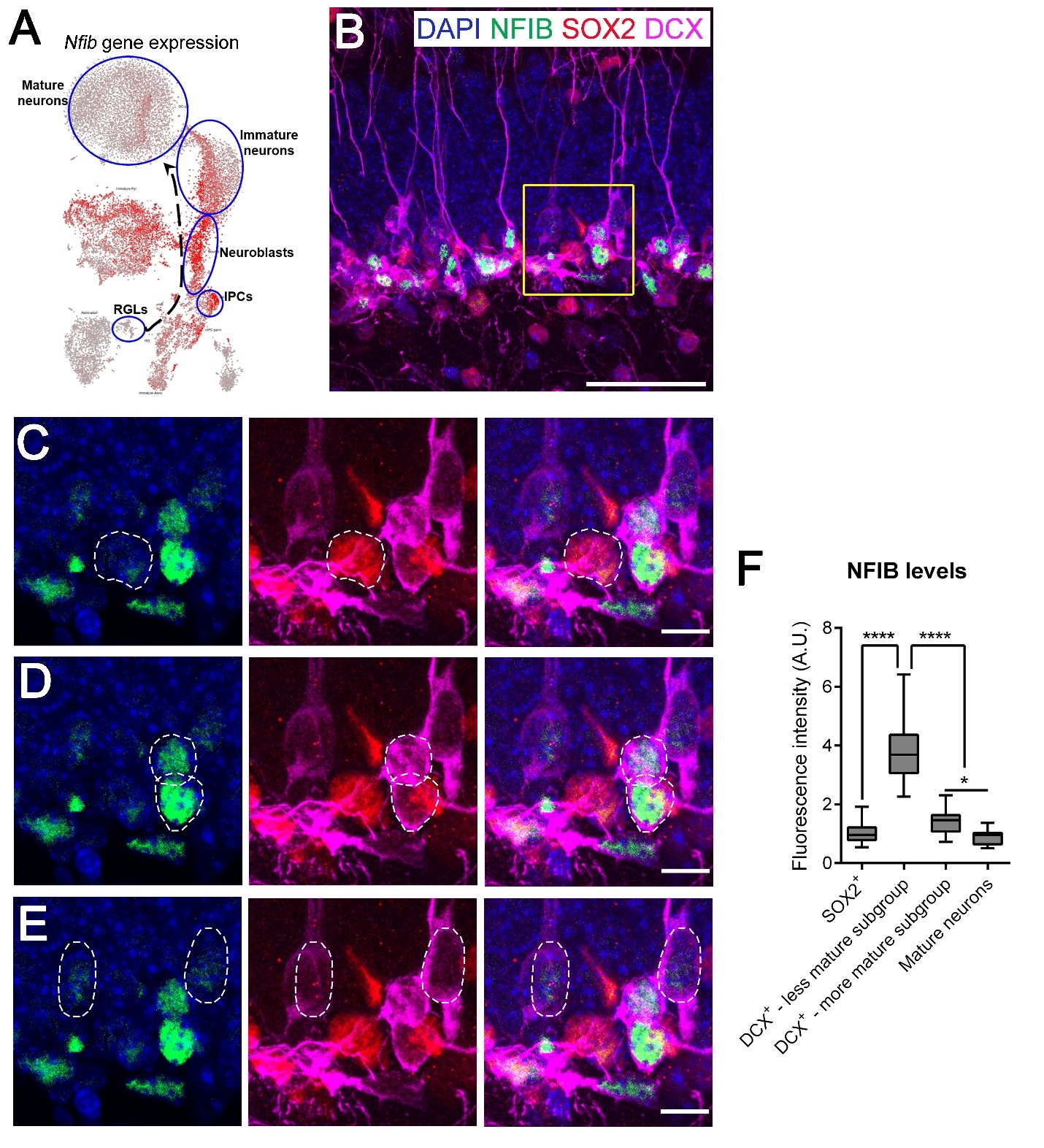
**

**Figure S10. Expression pattern of NFIB during adult hippocampal neurogenesis.** (**A**) tSNE plot of adult dentate gyrus single-cell RNA sequencing data showing *Nfib* gene expression along the neurogenic lineage. Highest mRNA levels of *Nfib* are found in neuroblasts. *Nfib* levels drop during neuronal maturation. The data is generated using an online browser from the Linnarsson lab (http://linnarssonlab.org/dentate/). (**B**) NFIB and cell stage-specific marker expression in the dentate gyrus of adult control mice, showing DAPI (blue), NFIB (green), SOX2 (red) and DCX (magenta) staining. Scale bar = 50 µm. (**C**) NFIB shows low expression in a SOX2^+^ neural progenitor cell. (**D**) Within the DCX^+^ cell population, strongest NFIB expression is found in the less mature subgroups, which are the ones without a radial process or with strong DCX expression and a small cell body. (**E**) NFIB level drops in the most mature subgroup of DCX^+^ cells, which have low DCX expression and a large cell body with elaborate dendrites. Scale bars in C-E = 10 µm. (**F**) Quantification of relative fluorescence intensity of NFIB immunostaining in different cell stages. N = 44 SOX2^+^ cells, 74 DCX^+^ cells from the less mature subgroups, 15 DCX^+^ cell from the most mature subgroup, and 30 mature neurons from three 11-week-old control mice. Data are presented in box-and-whisker plots, where centre lines represent medians, box limits represent interquartile ranges, and whiskers represent minimum to maximum data ranges. Statistical analysis was performed with one-way ANOVA with Tukey’s *post hoc* test. * *P* < 0.05; **** *P* < 0.0001.

**Figure S11**

**
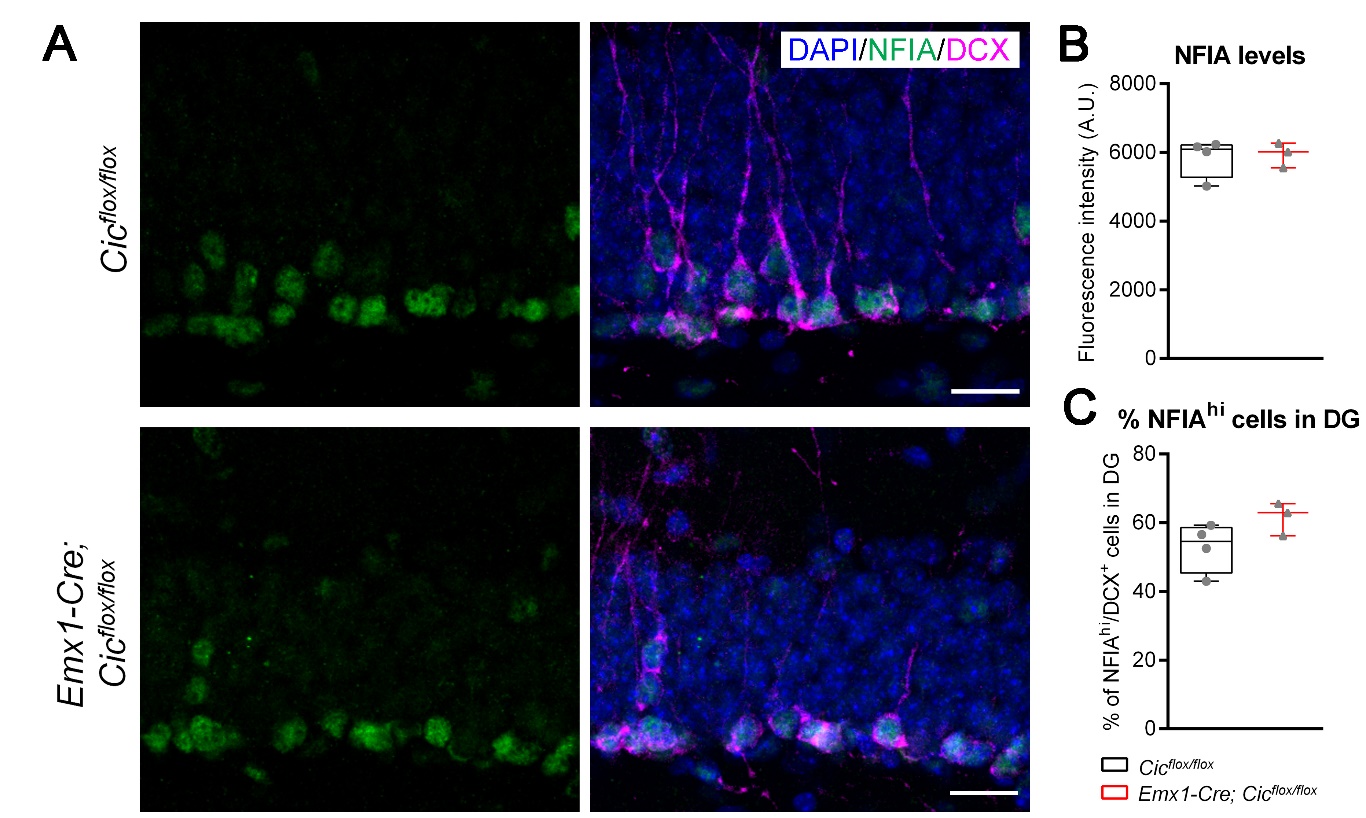
**

**Figure S11. NFIA levels in DCX^+^ cells are not altered in the *Emx1-Cre* *Cic* knockout mice.** (**A**) Representative images of DAPI (blue), NFIA (green) and DCX (magenta) staining in the dentate gyrus. The highest NFIA expression is found in DCX^+^ cells. Scale bars = 20 µm. (**B**) Quantification of fluorescent staining intensity of NFIA in neuroblasts. More than 50 cells per animal were analysed. Each data point represents the average value from multiple cells per animal. There is no difference between control and knockout mice. (**C**) Quantification of the percentage of DCX^+^ neuroblasts co-expressing NFIA. No significant difference is reported. N = 3-4 animals per group. Data are presented in box-and-whisker plots showing all data points, where centre lines represent medians, box limits represent interquartile ranges, and whiskers represent minimum to maximum data ranges. Statistical analysis was performed with one-way ANOVA with Tukey’s *post hoc* test.

**Figure S12**

**
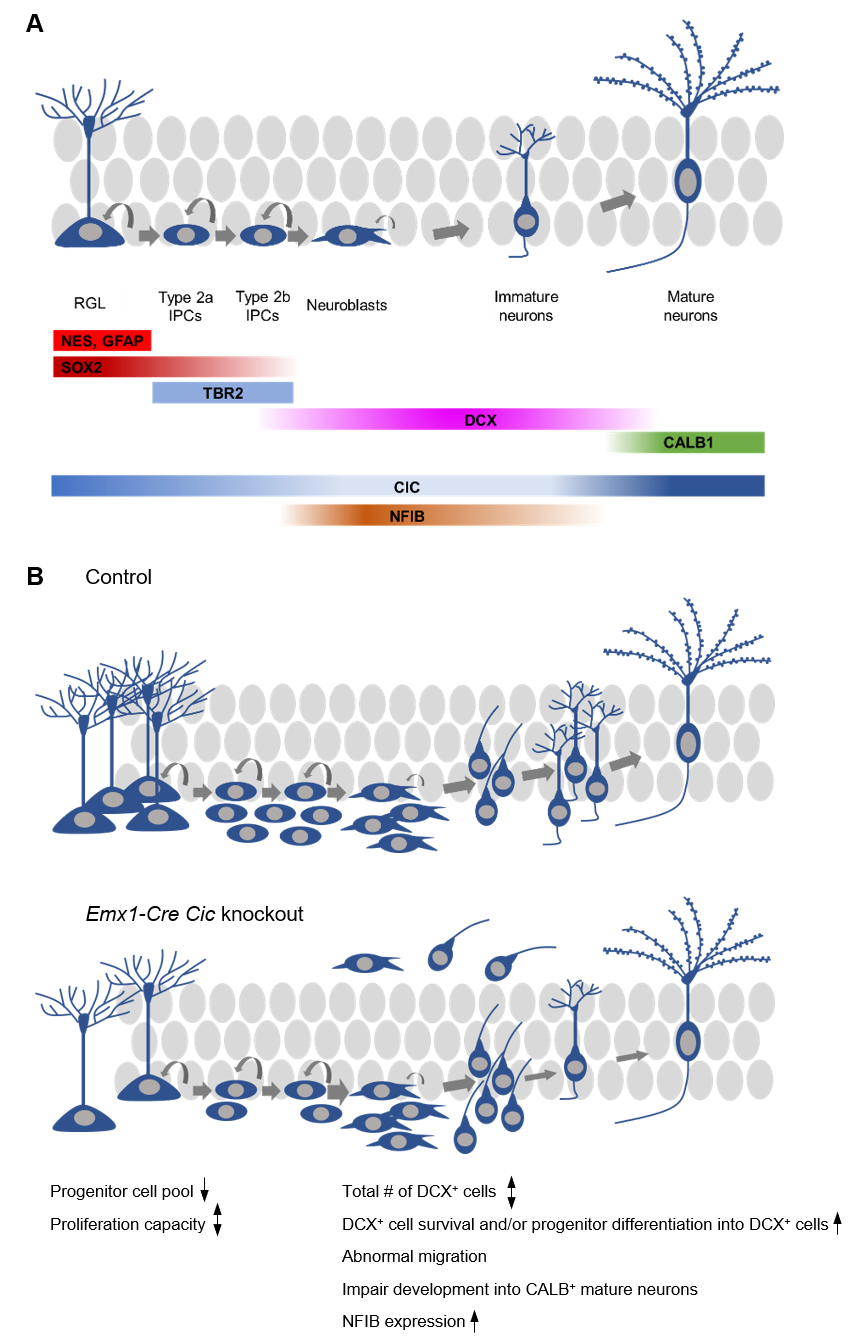
**

**Figure S12. Summary of key findings.** (**A**) A schematic of adult hippocampal neurogenesis highlights the complementary expression patterns of CIC and NFIB during granule neuron lineage development. (**B**) *Cic* deletion leads to a reduced adult neural progenitor cell pool and several defects in the DCX^+^ neuroblasts/immature neurons.
